# A single promoter‐TALE system for tissue‐specific and tuneable expression of multiple genes in rice

**DOI:** 10.1111/pbi.13864

**Published:** 2022-06-24

**Authors:** Florence Danila, Tom Schreiber, Maria Ermakova, Lei Hua, Daniela Vlad, Shuen‐Fang Lo, Yi‐Shih Chen, Julia Lambret‐Frotte, Anna S. Hermanns, Benedikt Athmer, Susanne von Caemmerer, Su‐May Yu, Julian M. Hibberd, Alain Tissier, Robert T. Furbank, Steven Kelly, Jane A. Langdale

**Affiliations:** ^1^ Australian Research Council Centre of Excellence for Translational Photosynthesis, Plant Sciences Division, Research School of Biology Australian National University Canberra Australian Capital Territory Australia; ^2^ Department of Cell and Metabolic Biology Leibniz Institute of Plant Biochemistry Halle Germany; ^3^ Department of Plant Sciences University of Cambridge Cambridge UK; ^4^ Department of Plant Sciences University of Oxford Oxford UK; ^5^ Biotechnology Center National Chung Hsing University Taichung Taiwan; ^6^ Institute of Molecular Biology Academia Sinica Taipei Taiwan; ^7^ Present address: Plant Breeding and Genetics Section, School of Integrative Plant Science Cornell University Ithaca New York USA

**Keywords:** synthetic gene circuits, rice, cell‐type‐specific gene expression, dTALE‐STAP

## Abstract

In biological discovery and engineering research, there is a need to spatially and/or temporally regulate transgene expression. However, the limited availability of promoter sequences that are uniquely active in specific tissue‐types and/or at specific times often precludes co‐expression of multiple transgenes in precisely controlled developmental contexts. Here, we developed a system for use in rice that comprises synthetic designer transcription activator‐like effectors (dTALEs) and cognate synthetic TALE‐activated promoters (STAPs). The system allows multiple transgenes to be expressed from different STAPs, with the spatial and temporal context determined by a single promoter that drives expression of the dTALE. We show that two different systems—dTALE1‐STAP1 and dTALE2‐STAP2—can activate STAP‐driven reporter gene expression in stable transgenic rice lines, with transgene transcript levels dependent on both dTALE and STAP sequence identities. The relative strength of individual STAP sequences is consistent between dTALE1 and dTALE2 systems but differs between cell‐types, requiring empirical evaluation in each case. dTALE expression leads to off‐target activation of endogenous genes but the number of genes affected is substantially less than the number impacted by the somaclonal variation that occurs during the regeneration of transformed plants. With the potential to design fully orthogonal dTALEs for any genome of interest, the dTALE‐STAP system thus provides a powerful approach to fine‐tune the expression of multiple transgenes, and to simultaneously introduce different synthetic circuits into distinct developmental contexts.

## Introduction

Engineering biology is becoming ever more ambitious as a discipline, aiming to create entirely synthetic pathways and/or to recapitulate evolution in a range of organisms. In plants, the manipulation of photosynthesis and nitrogen fixation pathways for crop improvement are just two examples of challenges that are underway (Bailey‐Serres *et al.,* 2019; Burén *et al.,* 2018; Ermakova *et al.,* 2020). A shared feature of many biological engineering challenges is the need to express multiple genes in a tissue‐specific manner, preferably at different levels, with limited off‐target effects. Tissue‐specificity can be accomplished with promoters that have the desired expression pattern (Butelli *et al.,* 2008; Dutt *et al.,* 2014; Grützner *et al.,* 2021; Tissier *et al.,* 2013) and if multiple promoters for the targeted tissue are available, multiplexed and tuned tissue‐specific gene expression might be possible via direct fusion of coding sequences to these promoters. However, because multiple promoters for desired developmental contexts are not always available and repeat use of the same promoter in a single transgene construct presents the risk of silencing and/or transcriptional squelching (Assaad *et al.,* 1993; Rajeev Kumar *et al.,* 2015), there is a need to develop alternative strategies for the simultaneous activation of pathway components.

One solution to the limited availability of tissue‐specific promoters is to use a well‐characterized promoter to drive expression of a transcriptional activator, which in turn can activate multiple cognate promoter sequences. In such a configuration, a single promoter with tissue‐specificity is required to achieve tissue‐specific expression of multiple transgenes. However, avoiding interference by the introduced circuit with endogenous pathways requires having recourse to orthogonal transcriptional activators. An additional desirable feature is the tunability of individual promoters, so that a complex response with desired stoichiometry and outcome can be achieved. A first incarnation of such a system in eukaryotic organisms was the yeast‐based GAL4/UAS system that was developed for use in mammalian cells, Drosophila, and plants (Brand and Perrimon, 1993; Haseloff, 1998; Kakidani and Ptashne, 1988). One constraint of GAL4, however, is that it binds a defined consensus sequence and as such only one synthetic circuit can be introduced into the genome. This precludes adoption for complex engineering tasks that require different circuits to operate in specific tissues or at specific development stages. This limitation was overcome with the advent of programmable DNA‐binding proteins such as Zinc Finger, CRISPR‐Cas (clustered regularly interspaced short palindromic repeats), or TALEs (transcription activator‐like effectors) (Piatek and Mahfouz, 2017).

**Figure 1:**
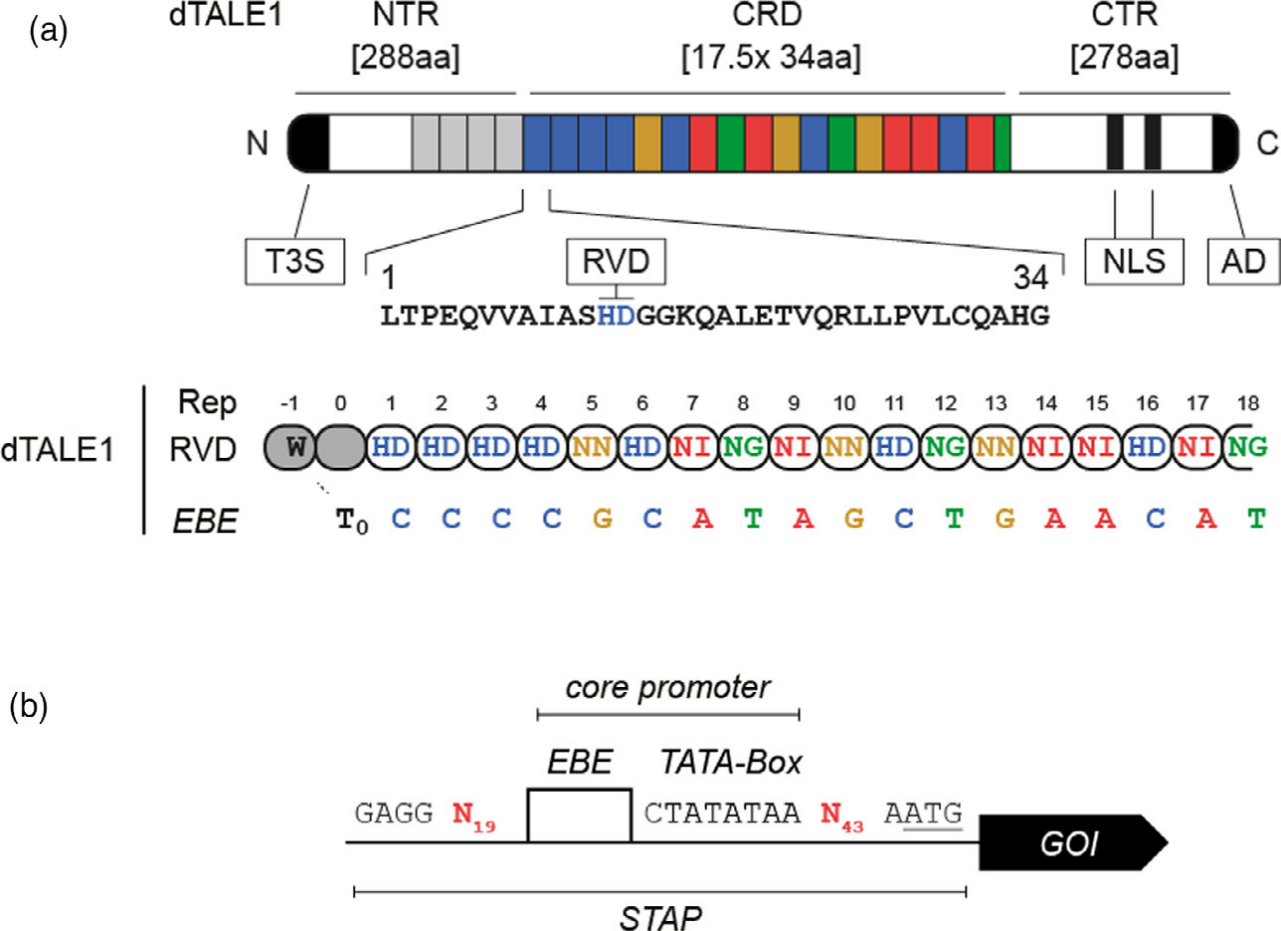
Application of TALEs as transcriptional regulators. (a) Schematic representation of the TALE DNA‐binding mode using dTALE1 as an example. TALEs specifically bind to DNA via their central repeat domain (CRD). The Repeat Variable Diresidue (RVD) of each repeat defines its binding specificity. TALE EBEs are naturally preceded by a 5′Thymine (T_0_). NTR—N‐terminal region (grey rectangles indicate degenerated repeats responsible for T_0_ coordination); CTR—C‐terminal region; T3S—type III secretion signal; NLS—nuclear localization signal; AD—acidic activation domain; Rep—repeat; EBE—effector‐binding element. (b) Schematic representation of the general STAP architecture. The STAP library was constructed as Golden Gate modules with flanking overhangs GGAG (5′) and AATG (3′). The common core element contains the EBE and a TATA‐box motif. The core promoter is flanked by variable 19 bp and 43 bp sequences upstream and downstream, respectively. Translational start (ATG) is underlined.

Systems that exploit synthetic CRISPR‐based transcriptional activators or reprogrammed natural TALEs have been reported, each with potential advantages and limitations. Bacterial‐derived CRISPR‐based RNA‐guided endonucleases can be applied to target specific loci within a genome by complementary spacer sequences of the guide RNA and the presence of an essential protospacer adjacent motif (PAM) (Jiang and Doudna, 2017; Nishimasu and Nureki, 2017). Catalytically dead endonucleases (e.g. Cas9(D10AH840A) – dCas9) can be used as a chassis for sequence‐specific RNA‐guided DNA‐binding, and by fusion or recruitment of transcription activator domains or epigenetic modifiers, Cas proteins can be converted into programmable transcriptional regulators (Guo *et al.,* 2022; Li *et al.,* 2017; Pan *et al.,* 2021; Papikian *et al.,* 2019; Shakirova *et al.,* 2020). By using several individually expressed sgRNAs or sgRNA arrays (multiplexing), a single Cas‐based transcriptional activator is sufficient to induce multiple target genes (Čermák *et al.,* 2017; Lowder *et al.,* 2018; Pan *et al.,* 2021; Xiong *et al.,* 2021). Similar approaches exploit bacterial‐derived TALEs which are naturally evolved transcriptional activators that bind to DNA in the nuclei of plant cells upon pathogen infection (Boch and Bonas, 2010). Specific TALE DNA‐binding occurs via a central modular DNA‐binding domain which consists of several tandemly arranged, nearly identical 34–35 amino acid long central repeat domains (CRDs) that differ at two positions (amino acids 12 and 13) called repeat‐variable diresidue (RVD) repeats (Figure 1a). TALE DNA‐binding occurs in a ‘one‐repeat to one base pair’ manner in which the RVD defines the binding specificity of a given repeat (Boch *et al.,* 2009; Moscou and Bogdanove, 2009). Naturally occurring RVDs have amino acid variants HD, NN, NI, or NG that mediate specific, modular binding to Cytosine, Guanine/Adenine, Adenine, and Thymine, respectively (Juillerat *et al.,* 2015; Miller *et al.,* 2015; Yang *et al.,* 2014). RVD‐defined target sequences (effector‐binding elements—EBEs) are almost always preceded by an invariant 5’ Thymine (T_0_) (Figure 1a; Doyle *et al.,* 2013; Gao *et al.,* 2012; Mak *et al.,* 2012; Schreiber and Bonas, 2014; Schreiber *et al.,* 2015). Together, these features provide a code which allows the design of synthetic TALEs with customized repeat (RVD)‐orders (called designer TALEs—dTALEs) that can bind DNA sequences of choice and that can be synthesized and assembled efficiently using a Golden Gate‐based cloning system (Engler *et al.,* 2014; Weber *et al.,* 2011; Weber *et al.,* 2011).

Recently, a library of small synthetic TALE‐activated promoters (STAPs) that contain minimal sequence requirements for dTALE‐mediated regulation was developed (Brückner *et al.,* 2015; Schreiber and Tissier, 2017) (Figure 1b). In this system, referred to as dTALE1‐STAP1, the core element that all STAP1s have in common is the dTALE1‐specific EBE followed by a TATA‐Box motif. The TATA‐Box was included because in host genes many natural TALE EBEs overlap or are located close to TATA‐box motifs of natural promoters (Grau *et al.,* 2013; Streubel *et al.,* 2017). In the dTALE1 system, the STAP1 core‐element is flanked by 19 bp and 43 bp variable sequences upstream and downstream, respectively. Quantitative transient reporter assays revealed that different EBE‐flanking sequences have variable effects on the strength of TALE‐mediated transcriptional induction (Brückner *et al.,* 2015; Streubel *et al.,* 2017), in principle allowing fine‐tuning of downstream gene expression strength by choosing corresponding STAPs (Brückner *et al.,* 2015). Whether such libraries are actually suitable for synthetic circuit applications needs to be tested in stable transgenic lines to determine whether genome insertion site impacts dTALE and/or STAP activity and whether synthetic dTALEs activate off‐target host gene expression.

The C_4_ Rice Project (https://c4rice.com/) aims to engineer C_4_ photosynthesis into the C_3_ plant rice. As this is a highly complex engineering endeavour that requires modified expression of multiple genes in several tissues, the implementation of an orthogonal transcriptional activation system could help accelerate project goals. Here, we tested the suitability of the dTALE1‐STAP1 system for biological engineering in rice and also designed and tested a second (dTALE2‐STAP2) system. The efficacy of both systems was evaluated by analysing the abundance and cellular localization of reporter gene transcripts in transgenic rice lines that were transformed with a multigene construct: dTALE1 or dTALE2 expression was driven by a cell‐type‐specific promoter and the expression of a single or multiple genes was driven from cognate STAPs. Genome‐wide transcriptome analysis was used to quantify transgene expression levels and to identify off‐target effects of dTALE activity on endogenous gene expression profiles. Collectively, these analyses demonstrate the suitability of the dTALE‐STAP system for engineering the expression of multiple transgenes, in precisely controlled cellular contexts, in stable transgenic rice lines.

## Results

### STAP‐driven reporter gene expression in stable transgenic lines is dTALE‐dependent, with the level of expression dependent on both dTALE and STAP sequence identity

**Figure 2:**
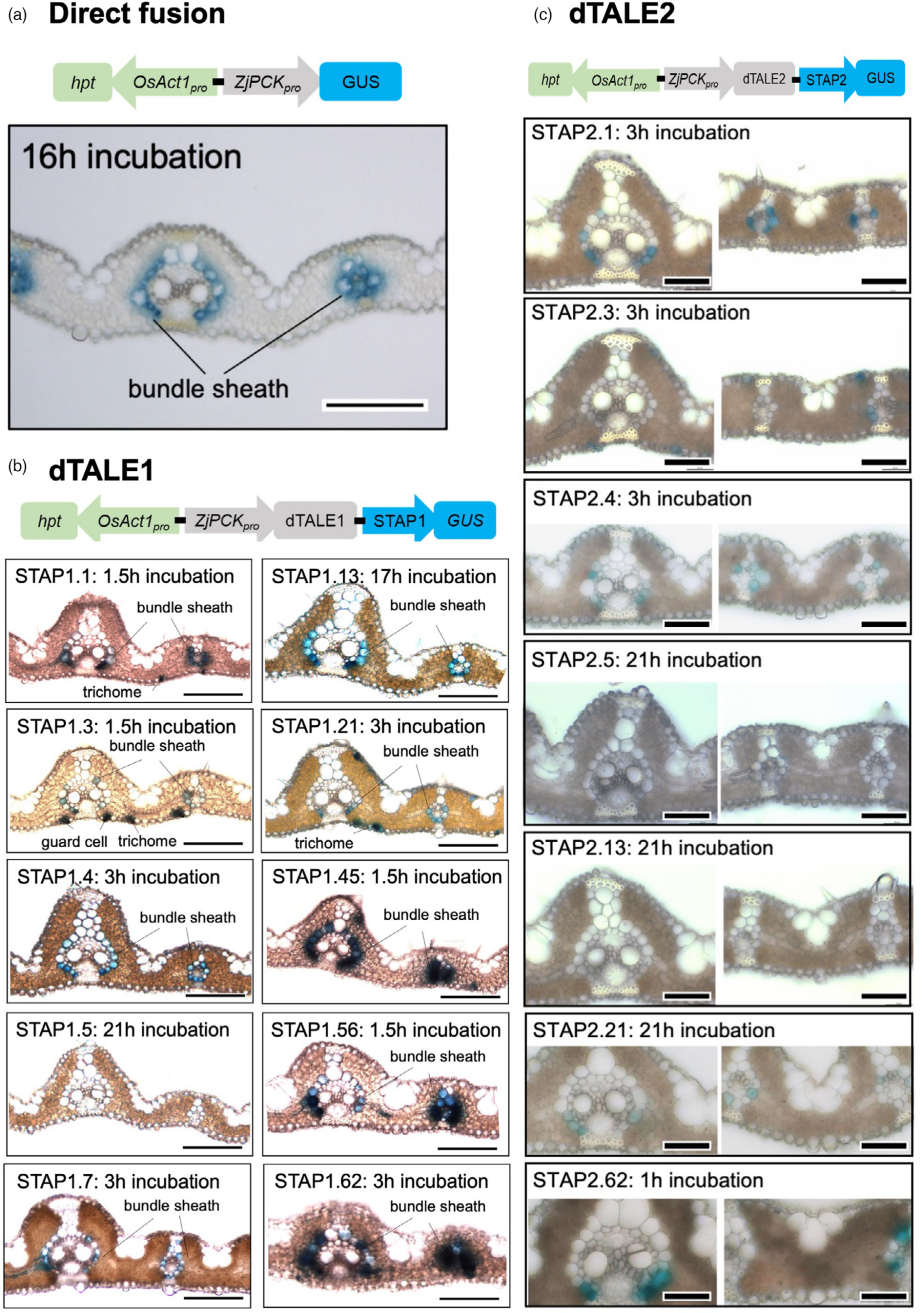
Cellular localization of GUS activity in bundle sheath cells of T_0_ transgenic lines. (a) GUS staining of bundle sheath cells in a transverse leaf section from a *ZjPCK*_*pro*_:GUS stable rice transformant. (b, c) GUS activity after staining at 37 °C for the indicated time period in transverse leaf sections of single insertion rice lines containing ten different *ZjPCK*_*pro*_:dTALE1‐STAP1:GUS constructs (b) and seven different *ZjPCK*_*pro*_:dTALE2‐STAP2:GUS constructs (c). Scale bars = 100 μm (a and b), 50 μm (c).

To assess the functionality of the dTALE‐STAP system in stable transgenic rice lines, the ability of STAPs to drive reporter gene expression in a dTALE‐dependent manner was evaluated. Both the original dTALE1‐STAP1 system and a second system designed to be orthogonal to the rice genome (dTALE2‐STAP2) (Methods and Figure S1) were tested. To compare the relative activity of the two dTALEs and of different STAPs in each system, plants were transformed with constructs in which dTALE expression was driven by the bundle sheath cell‐specific *Zoysia japonica PHOSPHOENOLPYRUVATE CARBOXYKINASE* (*ZjPCK)* promoter (*ZjPCK*_*pro*_) (Nomura *et al.,* 2005) and individual STAPs were fused to the GUS reporter gene. To act as a baseline for GUS activity level, rice lines with a direct *ZjPCK*_*pro*_:GUS fusion were also generated. GUS staining performed with leaves of the *ZjPCK*_*pro*_:GUS line showed activity of GUS after 16 h of staining (Figure 2a). By contrast, GUS activity was detected in most *ZjPCK*_*pro*_:dTALE1‐STAP1:GUS lines in <3 h (Figure 2b). Taking into account variability between independent transgenic lines that is likely caused by transgene position effects, the relative strength of individual STAP1s loosely grouped into four classes based on the time taken to detect qualitatively equivalent levels of GUS activity—inactive (STAP1.5), weak (STAPs 1.1, 1.3, 1.13), medium (STAPs 1.4, 1.21), and strong (STAPs 1.7, 1.45, 1.56, 1.62) (Table 1). The relative strength of individual STAPs was similar in the dTALE2‐STAP2 system, with STAP2.62 lines showing much higher levels of GUS activity than STAP 2.1, 2.3, 2.4, and 2.21 lines, and GUS activity being virtually undetectable in lines containing STAP 2.5 and 2.13 (Figure 2c, Table 2). However, overall levels of GUS activity were much lower in *ZjPCK*_*pro*_:dTALE2‐STAP2:GUS lines than in *ZjPCK*_*pro*_:dTALE1‐STAP1:GUS lines (Figure 2b, c). Control lines, in which dTALE1 was replaced by dsRed showed no GUS activity after a 21 h staining period (Figure S2). Collectively these results demonstrate that the dTALE‐STAP system is functional in stable transgenic lines, with STAP activity dependent on the presence of the cognate dTALE, and the level of STAP‐driven gene expression influenced by both dTALE and STAP sequence identities.

**Table 1:** Activity level and tissue specificity of β‐glucuronidase (GUS) within GUS positive single insertion T_0_ lines of the ten *ZjPCK*_*pro*_:dTALE1‐STAP1s analysed. BS = bundle sheath

STAP #.	1.1	1.3	1.4	1.5	1.7	1.13	1.21	1.45	1.56	1.62
# single insertion lines used for GUS staining	13	17	22	3	10	37	23	15	20	20
# positive lines that stained for GUS	9	10	13	0	7	25	15	10	13	17
Activity level distribution (%)										
High	22	10	8	0	57	24	13	70	85	71
Moderate	22	40	46	0	29	36	67	30	8	18
Low	56	50	46	0	14	40	20	0	7	11
Tissue specificity distribution (%)										
BS	0	0	15	0	0	12	0	40	31	12
Trichome	0	40	23	0	0	8	0	0	0	0
Guard cell	0	0	0	0	29	0	0	0	0	0
BS and trichome	100	50	62	0	42	80	100	40	69	59
Trichome and guard cell	0	10	0	0	0	0	0	0	0	0
BS, trichome, and guard cell	0	0	0	0	29	0	0	20	0	29

**Table 2:** Activity level and tissue specificity of β‐glucuronidase (GUS) within GUS positive single insertion T_0_ lines of the seven dTALE2‐STAP2s analysed under three different promoters. M = mesophyll, BS = bundle sheath

STAP #	2.1	2.3	2.4	2.5	2.13	2.21	2.62
Promoter 1: *ZjPCK*_*pro*_							
# single insertion lines used for GUS staining	3	8	5	7	6	18	6
# positive lines that stained for GUS	3	7	3	1	0	15	3
Activity level distribution (%)							
High	0	0	0	0	0	0	34
Moderate	34	71	66	0	0	33	33
Low	66	29	34	100	0	67	33
Tissue specificity distribution (%)							
BS	100	14	100	100	0	46	67
Guard cell	0	14	0	0	0	27	0
BS and guard cell	0	72	0	0	0	27	33
Promoter 2: *ZmPEPC321*_*pro*_							
# single insertion lines used for GUS staining	6	7	8	10	12	9	0
# positive lines that stained for GUS	5	7	7	9	10	3	0
Activity level distribution (%)							
High	75	100	29	89	70	100	0
Moderate	25	0	42	11	10	0	0
Low	0	0	29	0	20	0	0
Tissue specificity distribution (%)							
M	0	14	29	11	20	0	0
M and BS	20	43	14	0	20	0	0
M, BS, and vasculature	40	14	14	0	0	0	0
M, BS, and guard cell	20	29	0	0	0	0	0
M, BS, vasculature, and guard cell	20	0	43	89	60	100	0
Promoter 3: *SvPEPC500*_*pro*_							
# single insertion lines used for GUS staining	5	5	9	5	13	4	5
# positive lines that stained for GUS	5	4	5	4	13	4	4
Activity level distribution (%)							
High	100	100	40	75	46	50	100
Moderate	0	0	40	0	15	50	0
Low	0	0	20	25	39	0	0
Tissue specificity distribution (%)							
M	0	0	40	25	46	0	0
M and BS	0	0	0	0	15	50	0
M, BS, and vasculature	20	0	0	0	15	50	0
M, BS, and guard cell	0	0	0	0	0	0	0
M, BS, vasculature, and guard cell	80	100	60	75	24	0	100

### STAP‐driven reporter gene expression can be spatially regulated using cell‐specific promoters to drive dTALE expression

To assess the cell‐specificity of reporter gene expression in the *ZjPCK*_*pro*_:dTALE‐STAP lines, localization of GUS activity was compared in transverse leaf sections of T_0_ lines transformed with the direct *ZjPCK*_*pro*_:GUS fusion (Figure 2a), *ZjPCK*_*pro*_:dTALE1‐STAP1:GUS (Figure 2b, Table 1) or *ZjPCK*_*pro*_:dTALE2‐STAP2:GUS (Figure 2c, Table 2) constructs. Analysis of T_0_ *ZjPCK*_*pro*_:dTALE1‐STAP1:GUS lines revealed that STAPs 1.1, 1.21, 1.45, 1.56, and 1.62 were the most consistent in terms of cell‐specificity, driving GUS activity in bundle sheath cells in multiple independent lines, although in some cases expression was also observed in trichomes and guard cells (Figure 2b, Table 1). GUS activity was similarly localized to bundle sheath and/or guard cells in *ZjPCK*_*pro*_:dTALE2‐STAP2:GUS lines (Table 2). Expression in trichomes and guard cells was not observed with the direct *ZjPCK*_*pro*_:GUS fusion (Figure 2a), however, the PCK promoter has previously been shown to be active at low levels in both of these cell‐types (Penfield *et al.,* 2012). Expression in these cell types in the dTALE‐STAP lines therefore likely reflects faithful amplification of *ZjPCK*_*pro*_ activity as opposed to ectopic expression.

**Figure 3:**
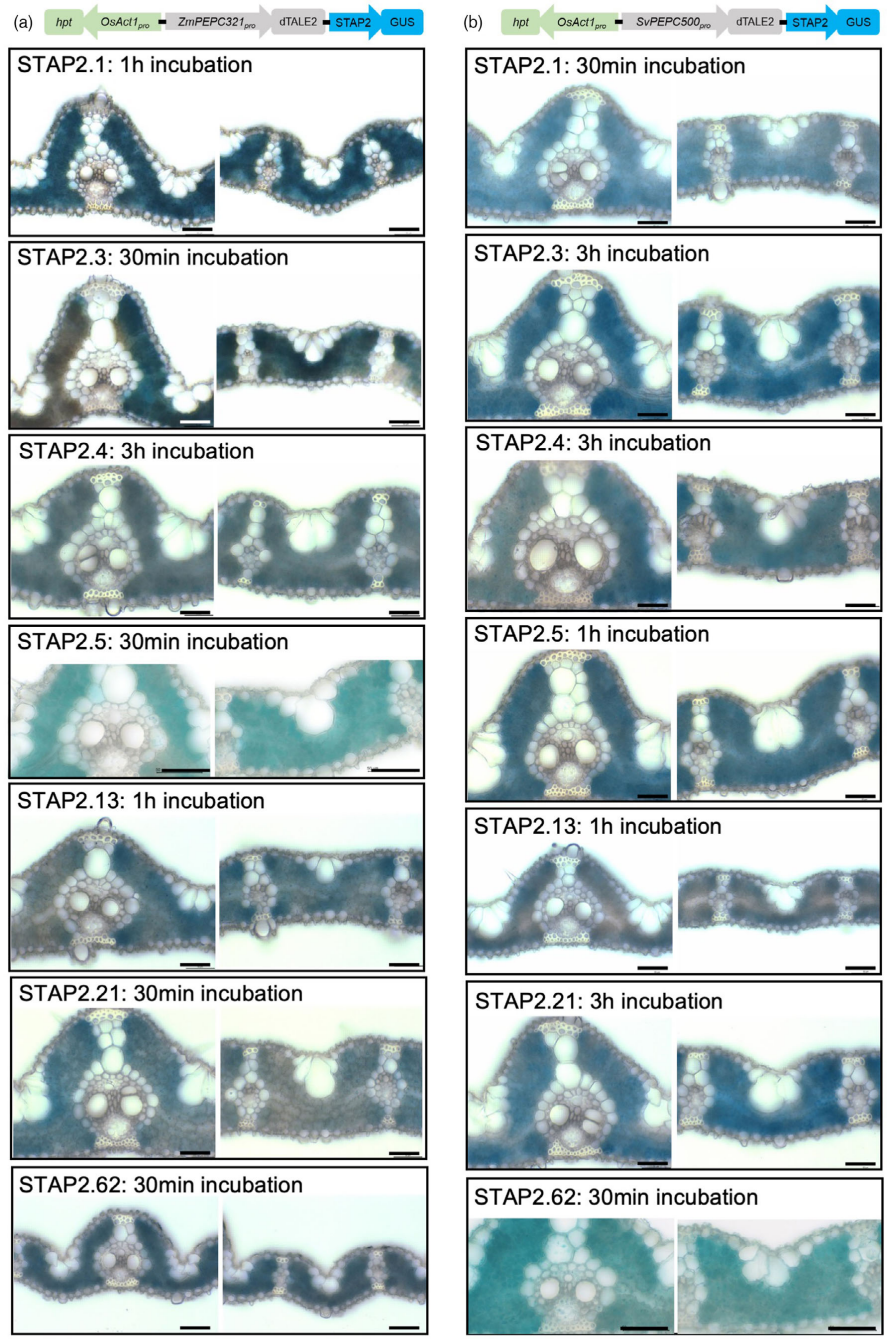
Cellular localization of GUS activity in mesophyll cells of T_0_ transgenic lines. (a) *ZmPEPC321*_*pro*_:dTALE2‐STAP2:GUS. (b) *SvPEPC500*_*pro*_:dTALE2‐STAP2:GUS. Staining was carried out at 37 °C for the indicated time period. Lines contained seven different STAP sequences as indicated and all contained a single transgene insertion (with the exception of *SvPEPC500*_*pro*_:dTALE2‐STAP2.62:GUS for which copy number was not determined). Scale bars = 50 μm.

To further investigate the effectiveness of combining cell‐specific promoters with the dTALE‐STAP system, two *PHOSPHOENOLPYRUVATE CARBOXYLASE* promoters (*PEPC*_*pro*_) known to direct strong mesophyll‐specific gene expression in rice were tested with the dTALE2‐STAP2 system. Expression of dTALE2 from *PEPC* promoters of either *Setaria viridis* (*SvPEPC500*_*pro*_) (Gupta *et al.,* 2020) or *Zea mays* (*ZmPEPC321*_*pro*_) (Gupta *et al.,* 2020; Matsuoka *et al.,* 1994) led to high levels of STAP2‐driven GUS activity, with many lines requiring just 30 min of staining for detection (Figure 3). Although activity was primarily detected in mesophyll cells, high levels of activity were occasionally associated with product detection in all cell types, most likely as a result of diffusion (Figure 3; Table 2). None of the lines transformed with negative control constructs (mTurquoise in place of dTALE2) showed GUS activity after a 21 h staining period (Figure S3). Of the seven STAPs assessed, STAPs 2.1, 2.3, 2.5, and 2.62 drove high levels of expression in over 75% of the *SvPEPC500*_*pro*_ and *ZmPEPC321*_*pro*_ lines tested (note, however, that STAP2.62 was only tested with *SvPEPC500*_*pro*_) (Table 2). Although direct comparisons are difficult in this qualitative framework, it is notable that unlike the high level of activity observed in mesophyll cells, when *ZjPCK*_*pro*_ was used to drive dTALE1 or dTALE2 expression in bundle sheath cells, STAPs 1.5 and 2.5 were inactive and STAPs 1.1/2.1 and 1.3/2.3 were very weak Collectively these results demonstrate that the cell‐specificity of STAP‐driven reporter gene expression faithfully replicates activity of the promoter used to drive dTALE expression, and that the strength of individual STAP promoter sequences differs in different cell‐types.

### Heritable cell‐specific expression of multiple genes can be driven by the dTALE‐STAP system in rice

**Figure 4:**
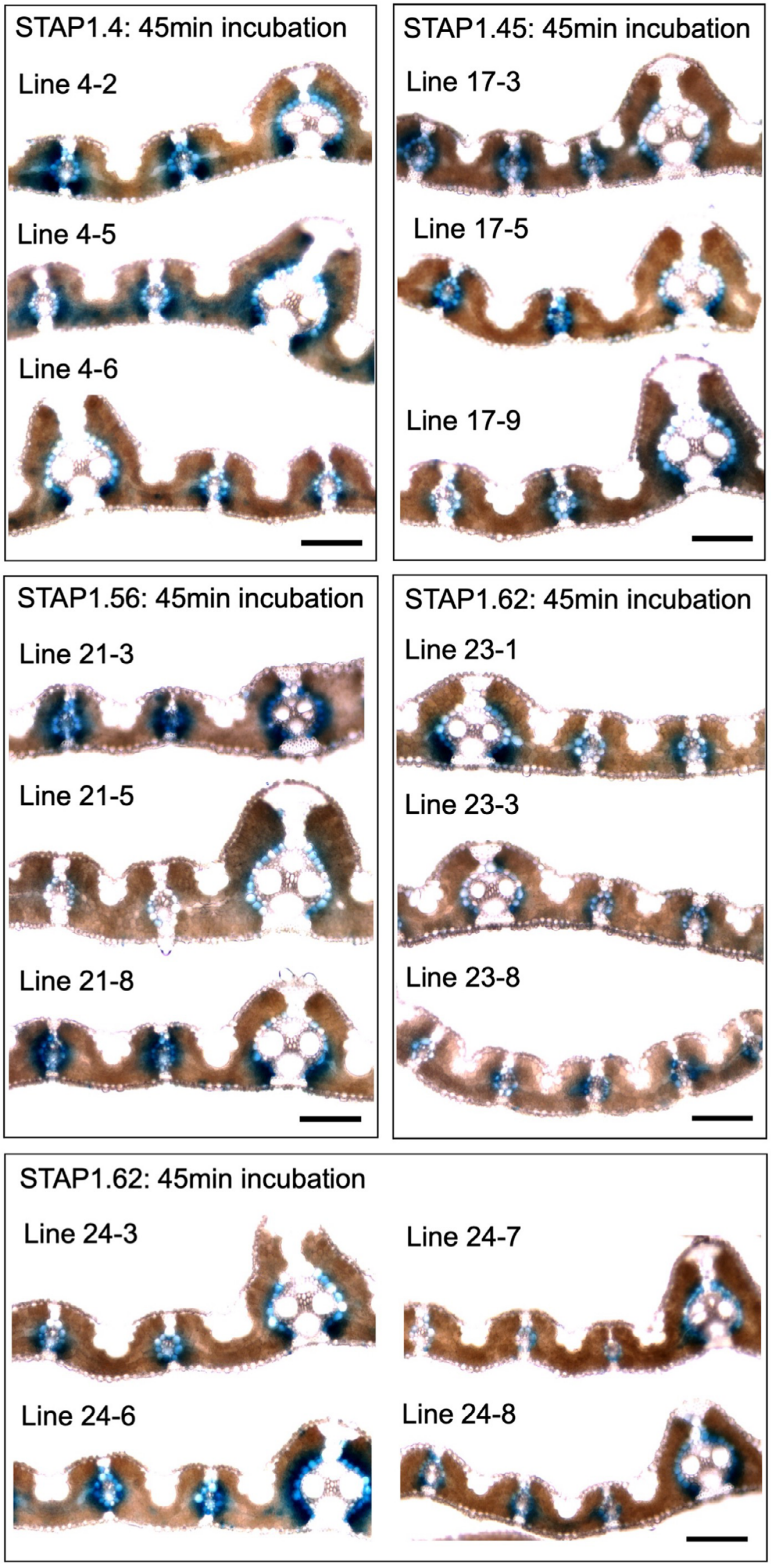
Cellular localization of GUS activity in T_1_ transgenic lines. All lines contain *ZjPCK*_*pro*_:dTALE1‐STAP1:GUS constructs. For STAP1.4, 1.45 and 1.56, lines are derived from a single independent insertion event whereas two independent insertion events are represented for STAP1.62. Scale bars = 100 μm.

**Figure 5:**
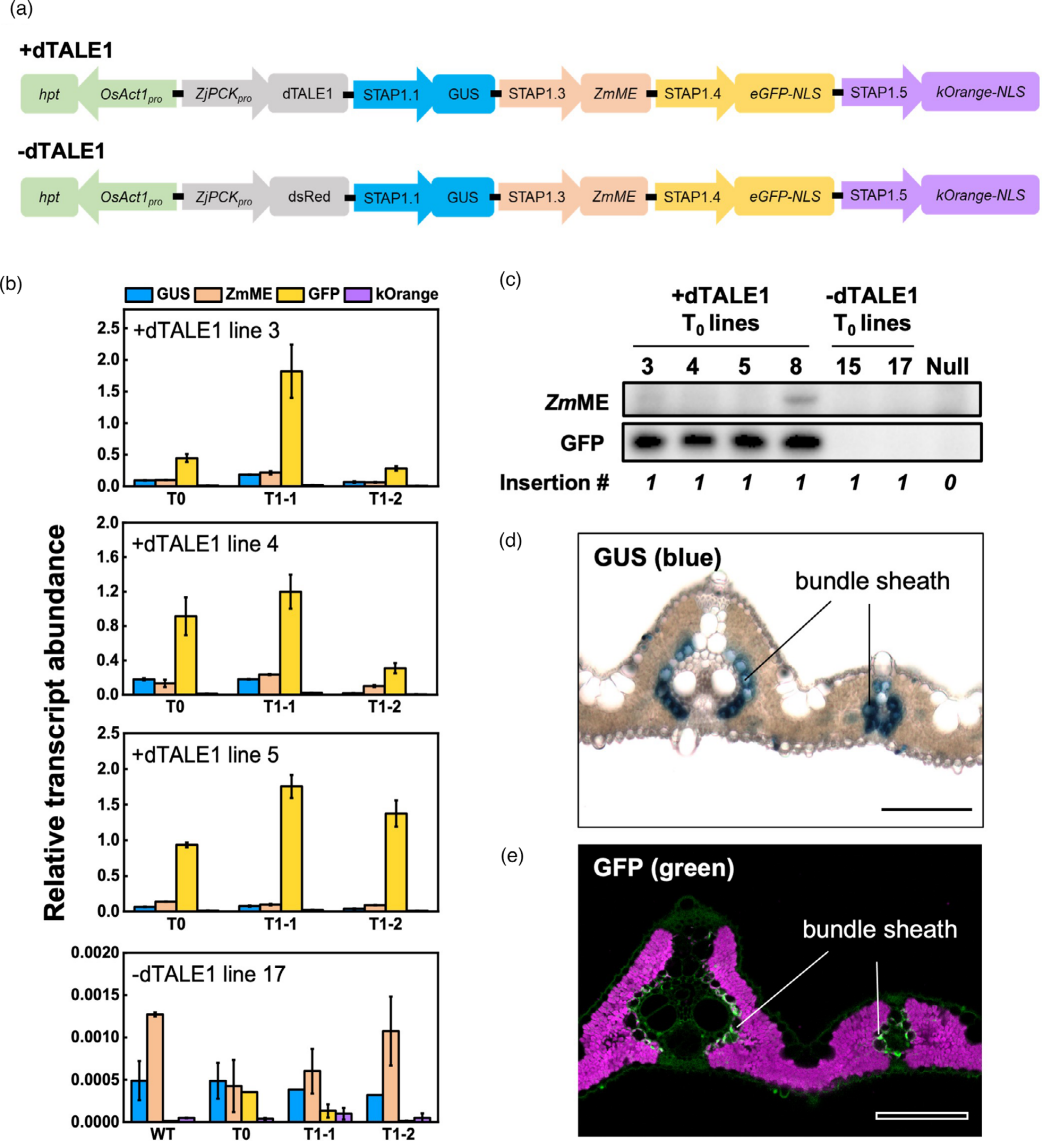
Tuneable and tissue‐specific expression of multiple genes in rice using the dTALE1‐STAP1 system. (a) Schematics of multi‐reporter gene constructs with and without dTALE1. (b) Relative transgene transcript levels in three single insertion T_0_ plants transformed with the +dTALE1 construct and one single insertion T_0_ plant transformed with the ‐dTALE1 construct, and in two homozygous plants from the respective T_1_ progeny. Very low levels of mis‐priming were seen with GUS and *Zm*ME primers in both WT controls and −dTALE1 lines. Mean ± SE, *n* = 3 technical replicates. (c) Accumulation of *Zm*ME and GFP proteins detected by western blotting in single insertion T_0_ plants with the +dTALE1 or ‐dTALE1 construct, and in azygous (null) plants. Insertions correspond to the *hpt* copy number in the plant. (d, e) Transverse leaf section of +dTALE1 T_0_ line 4 showing bundle sheath‐specific accumulation of GUS (d), and GFP (e). Fluorescence signals in (e) are pseudo‐coloured: green—GFP; magenta—chlorophyll autofluorescence. Bars = 100 μm.

To test the heritability of dTALE12‐STAP1 driven cell‐specific expression, GUS activity was analysed in T_1_ progeny of four *ZjPCK*_*pro*_:dTALE1‐STAP1:GUS lines that showed high levels of bundle sheath‐specific GUS activity in T_0_ plants (STAPs 1.4, 1.45, 1.56, 1.62). Notably, consistently high bundle sheath‐specific GUS activity was observed in all cases, requiring only 45 min of staining (Figure 4). These results suggest that expression profiles observed in T_0_ plants are inherited in T_1_ progeny. To determine whether multiple genes can be simultaneously activated by dTALE1 in a heritable manner, constructs with and without the dTALE1 transcription factor were generated to drive the expression of four reporter genes fused to STAP1s of different strengths (STAP1.1‐GUS, STAP1.3‐*Zm*ME, STAP1.4‐GFP‐NLS, and STAP1.5‐kOrange‐NLS; Figure 5a). qRT‐PCR using RNA extracted from T_0_ and T_1_ transgenic lines showed that, consistent with single STAP1‐GUS staining results in bundle sheath cells (Table 1, Figure 2b), STAP1.4 generated high levels of GFP transcript, whereas STAP1.5 drove very low levels of kOrange gene expression, with STAP1.1. and STAP1.3 activity levels in between (Figure 5b). Immunoblotting similarly revealed higher levels of STAP4‐driven GFP than STAP3‐driven ME protein (Figure 5c). Importantly, qRT‐PCR with lines containing dsRed instead of dTALE1 revealed levels of GUS, *Zm*ME, GFP, and kOrange transcripts that were indistinguishable from wild‐type controls (Figure 5b). Notably, transcript levels for each of the reporter genes in the multigene construct were consistent through two generations, demonstrating heritability of dTALE1‐mediated multigene activity (Figure 5b). Furthermore, consistent with use of the *ZjPCK*_pro_ to drive dTALE1 expression, protein localization (where detectable) was specific to bundle sheath cells (Figure 5d‐e). In summary, the dTALE1‐STAP1 system can be used to activate multiple genes in a specific cell‐type in a heritable manner.

**Figure 6:**
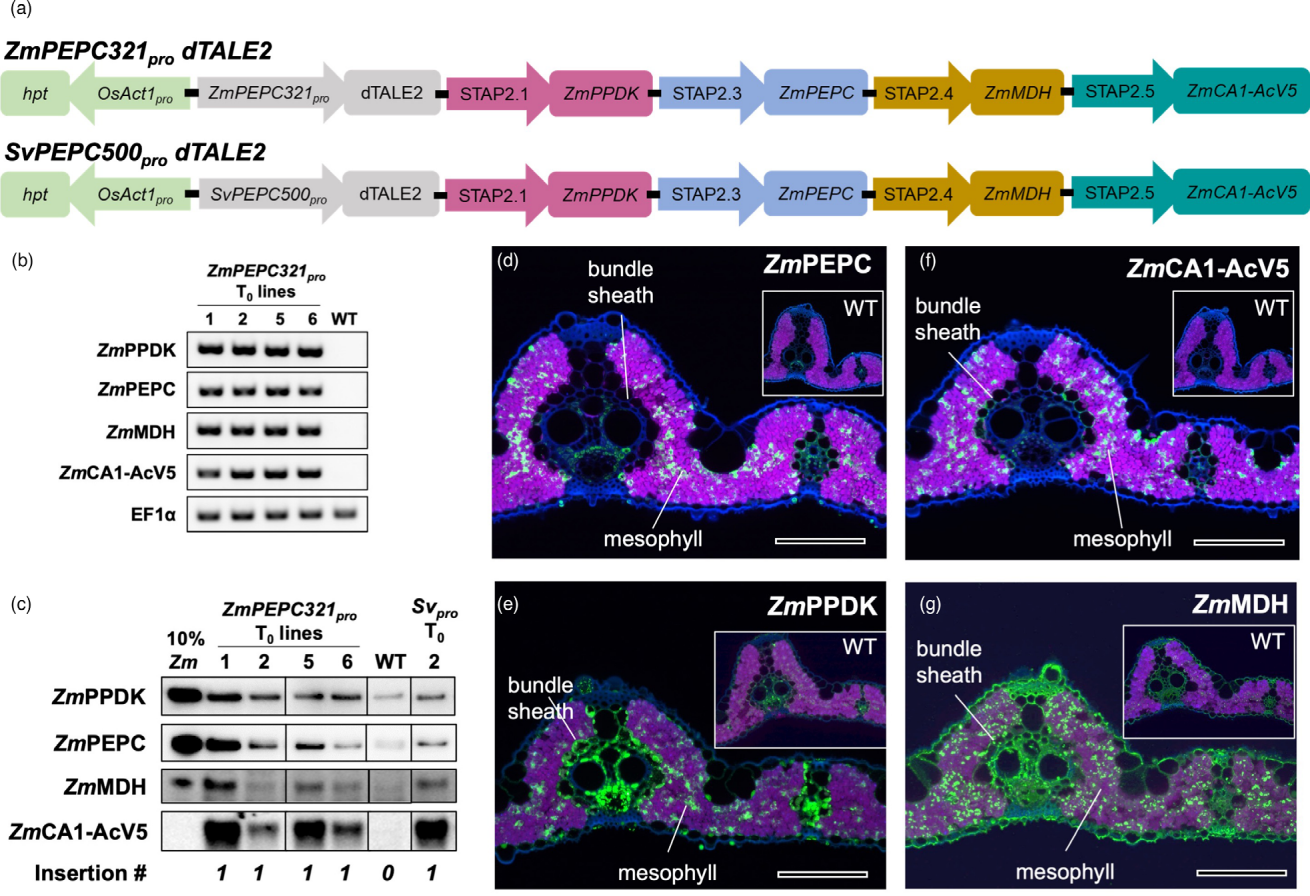
Tuneable and tissue‐specific expression of multiple genes in rice using the dTALE2‐STAP2 system. (a) Schematics of dTALE2‐STAP2 multigene constructs used for rice transformation. dTALE2 is expressed from the mesophyll cell‐specific promoters *ZmPEPC321*_*pro*_ or *SvPEPC500*_*pro*_. Four different STAP2s drive expression of the maize *ZmPPDK*, *ZmPEPC*, *ZmMDH,* and *ZmCA1* genes. *ZmCA1* is translationally fused to an AcV5 tag. (b) RT‐PCR showing transgene expression in single insertion *ZmPEPC321*_*pro*_ dTALE2 T_0_ lines but not in wildtype (WT). Elongation Factor (EF1α) was used as a positive control. (c) Protein accumulation detected by immunoblotting in single insertion T_0_ rice lines containing either *ZmPEPC321*_*pro*_ or *SvPEPC500*_*pro*_ (*Sv*_pro_) constructs, as compared to WT. A 1/10 dilution of an equivalent protein extract from wild‐type maize (10% *Zm*) was used as a positive control and wild‐type rice (WT) as a negative control. Note some cross‐reaction to endogenous rice enzymes with the PPDK, PEPC and MDH antibodies but not with the AcV5 tag antibody. Transgene insertion # corresponds to the *hpt* copy number detected by digital drop PCR. D‐G) Transverse leaf sections of *ZmPEPC321*_*pro*_ dTALE2 T_0_ line 5 showing accumulation of PEPC (d), PPDK (e), CA (f), and MDH (g) proteins in mesophyll cells. Insets show cross reactivity to endogenous PPDK (e) and MDH (g) proteins in WT, and to a lesser extent to PEPC (d), particularly in vascular associated tissues. Fluorescence signals are pseudo‐coloured: green—protein of interest labelled with secondary antibodies conjugated with AlexaFluor 488; magenta—chlorophyll autofluorescence; blue—calcofluor white‐stained cell walls. Bars = 100 μm.

Given the differences in relative STAP strengths observed between bundle sheath and mesophyll cells (e.g. STAP2.5 is inactive in bundle sheath cells but is very active in mesophyll cells), we further evaluated a dTALE2‐STAP2 multigene construct in which dTALE2 expression was driven in mesophyll cells by either *ZmPEPC321*_*pro*_ or *SvPEPC500*_*pro*_. Four STAP sequences that drove high (STAPs 2.1, 2.3, and 2.5) or moderate (STAP2.4) levels of mesophyll‐specific GUS expression in single *PEPC*_*pro*_:dTALE2‐STAP2:GUS constructs (Figure 3, Table 2) were tested. STAPs were fused to genes from *Zea mays* that encode the photosynthetic enzymes PEPC, pyruvate phosphate dikinase (PPDK), malate dehydrogenase (MDH), and carbonic anhydrase (CA). The strongest STAPs were fused to *ZmPPDK*, *ZmPEPC* and *ZmCA*, and *ZmCA* was translationally fused to an AcV5 tag (Figure 5a). RT‐PCR of T_0_ *ZmPEPC321*_*pro*_ *‐*dTALE2‐STAP2 lines revealed high transcript levels for all four transgenes (Figure 6b). Immunoblotting using antibodies against the maize proteins or the AcV5 tag, further revealed that all four proteins were clearly detectable in both *ZmPEPC321*_*pro*_ *‐*dTALE2‐STAP2 and *SvPEPC500*_*pro*_ *‐*dTALE2‐STAP2 T_0_ lines (Figure 6c), and immunolocalization confirmed protein accumulation in mesophyll cells (Figure 6d‐g). Collectively, these data demonstrate that relative STAP strength in multigene constructs can be predicted after empirical evaluation in single gene constructs, that both dTALE1‐STAP1 and dTALE2‐STAP2 systems can be used for the expression of multiple genes in a single transgenic construct, and that the expression of genes encoding both reporter proteins and functional enzymes can be achieved in a cell‐type‐specific context.

### STAP‐driven gene expression levels are amplified by dTALE1 but not dTALE2

**Figure 7:**
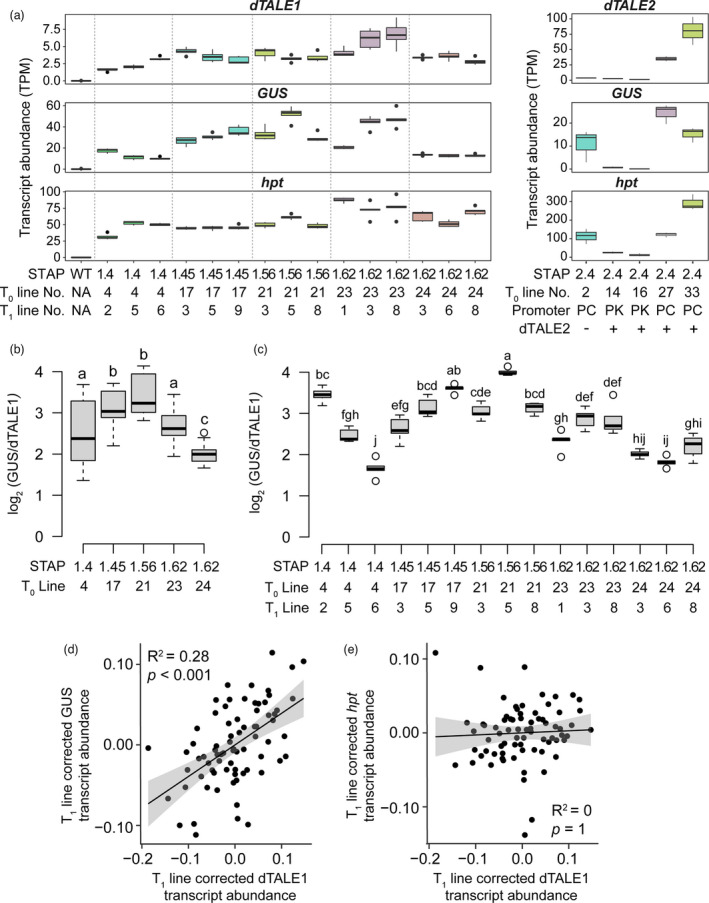
Amplification of transcript abundance with the dTALE/STAP system. (a) Transcript abundance of transgenes encoded on the *ZjPCK*_*pro*_:dTALE1‐STAP1:GUS, *ZjPCK*_*pro*_:dTALE2‐STAP2:GUS, and *ZmPEPC*_*pro*_:dTALE2‐STAP2:GUS constructs in stable transgenic lines. PC = *ZmPEPC*_*pro*_, PK = *ZjPCK*_*pro*_. (b, c) Variance in GUS/dTALE1 transcript abundance ratios between different STAPs in T_0_ (b) and T_1_ (c) *ZjPCK*_*pro*_:dTALE1‐STAP1:GUS lines. Letters above box plots indicate significant differences between groups (*P* < 0.05) from one‐way analysis of variance with Tukey test for multiple comparison. d) Correlation between GUS transcript abundance and dTALE1 transcript abundance in *ZjPCK*_*pro*_:dTALE1‐STAP1:GUS lines. (e) Correlation between *hpt* transcript abundance and dTALE1 transcript abundance in *ZjPCK*_*pro*_:dTALE1‐STAP1:GUS lines.

To quantify the amplification of GUS activity that was apparent in qualitative assays with dTALE1‐STAP1 (Figure 2, Table 1), transgene transcript levels were determined by RNA‐Seq. Transcriptomes were generated for single insertion T_1_ lines containing *ZjPCK*_*pro*_:dTALE1‐STAP1:GUS, *ZjPCK*_*pro*_:dTALE2‐STAP2:GUS, *ZmPEPC321*_*pro*_:dTALE2‐STAP2:GUS and *ZmPEPC321*_*pro*_:mTurquoise‐STAP2:GUS constructs. The lines sampled represented 10 independent transgene insertion sites (five for dTALE1, four for dTALE2, and one for the negative mTurquoise control) and four different STAPs (three unique to dTALE1–STAP1.45, 1.56, 1.62, and one common to both dTALE1 and dTALE2 – STAP1.4/2.4). We first sought to identify any influence of genomic context on transgene expression by quantifying the abundance of transcripts encoding the hygromycin resistance gene (*hpt*), which was driven by the ubiquitously active *OsAct1*_*pro*_. Transcript levels ranged between 6 and 340 transcripts per million (TPM), with significantly higher *hpt* levels in the two *ZmPEPC321*_*pro*_:dTALE2‐STAP2:GUS lines and the *ZmPEPC321*_*pro*_:mTurquoise‐STAP2:GUS line than in the five *ZjPCK*_*pro*_:dTALE1‐STAP1:GUS and two *ZjPCK*_*pro*_:dTALE2‐STAP2:GUS lines. Notably, GUS transcript levels were also elevated above baseline in the absence of the dTALE in the *ZmPEPC321*_*pro*_:mTurquoise‐STAP2.4:GUS line (Figure 7a). These data suggest that transgene transcript levels in the *ZmPEPC321*_*pro*_ lines were enhanced either by elements in the *ZmPEPC321*_*pro*_ itself or by genomic context.

To determine the level of dTALE expression in each line, total transcript levels were first evaluated. Transcript abundance ranged between 1.1 and 9.2 TPM when either dTALE1 or dTALE2 were expressed specifically in bundle sheath cells under the control of *ZjPCK*_*pro*_ and between 34 and 100 TPM when dTALE2 was expressed in mesophyll cells under the control of *ZmPEPC321*_*pro*_ (Figure 7a). Given that bundle sheath cells comprise around 10% of the total leaf area and mesophyll cells around 45% (Figure S4), we would expect dTALE transcript levels in lines with the mesophyll‐specific *ZmPEPC321*_*pr*o_ driving expression to be 4.5‐fold higher than those in lines with the bundle sheath specific *ZjPCK*_*pro*_, if the two promoters were of equivalent strength on a per cell basis. The data reveal up to 30‐fold higher levels of dTALE2 transcripts in *ZmPEPC321*_*pro*_ than *ZjPCK*_*pro*_ lines, suggesting that even if the aforementioned enhancement of transgene expression in the *ZmPEPC321*_*pro*_:dTALE2‐STAP2:GUS lines results from genomic context, on a per cell basis *ZmPEPC321*_*pro*_ drives much higher levels of dTALE expression than *ZjPCK*_*pro*_.

To compare the amplification capacity of dTALE1 and dTALE2, STAP‐driven GUS transcript abundance was evaluated in lines where dTALE expression was driven by the bundle sheath‐specific *ZjPCK*_*pro*_. This promoter was chosen instead of the stronger *ZmPEPC321*_*pro*_ so that any weak amplification effects could be detected. With dTALE1, the abundance of transcripts encoding GUS ranged between 8.6 and 59.9 TPM (Figure 7a), which is on average ~ 7.5‐fold higher than transcripts encoding dTALE1. By contrast, GUS transcript levels in dTALE2 lines ranged between 0.04 and 1.2 TPM, which is 11‐fold lower than dTALE2 transcript levels in the same lines. Thus, dTALE1 amplifies expression from the STAPs relative to its own expression level, whereas dTALE2 does not.

To quantify the effect of individual STAP sequences on transgene expression, the abundance of GUS transcripts was interrogated as a function of dTALE1 transcript abundance, STAP number, and transgene insertion site. In T_0_ lines containing STAP1.4, 1.45, 1.56, and 1.62, small but significant differences were seen in the GUS:dTALE1 ratio between different STAPs (Figure 7b). A similar extent of variation was observed in T_1_ lines (Figure 7c) both between individual T_1_ lines derived from the same T_0_ progenitor (STAPs 1.4, 1.45, and 1.56) and between T_1_ lines generated from independent T_0_ events (STAP 1.62). Thus, some variation of GUS:dTALE1 ratio was independent of transgene insertion site. A linear regression analysis was conducted to determine what proportion of variance in GUS abundance was attributable to different factors (Figure S5). The largest single component of variance in GUS expression was attributable to the homozygous T_1_ plant from which subsequent T_2_ plants were isolated (*R* ^2^ = 0.95, *P* < 2.2e‐16, Figure S5C). Notably, variation between different T_1_ lines descended from any single T_0_ transgene insertion event most likely resulted from tissue culture‐induced somatic mutations that either segregated independently and/or were inherited according to the position of the T_1_ seed on the inflorescence of the regenerated T_0_ plant. Correcting for this effect revealed that there was a significant association between the transcript abundance for GUS and the transcript abundance for dTALE1 (Figure 7d; *R* ^2^ = 0.28, *P* = 6.276e‐07, Figure S5E). In contrast, there was no association between the transcript abundance of dTALE1 and transcript abundance of the *OsAct1*_*pro*_‐driven *hpt* gene that is encoded on the same transgene construct (Figure 7e; *R* ^2^ = 0.036, *P* = 0.05687, Figure S5F). Thus, although there were substantial differences in transgene abundance between T_1_ lines, a significant interaction between the abundance of dTALE1 and GUS transcripts was observed, consistent with the proposed regulatory function of dTALE1. Furthermore, STAPs 1.4, 1.45, 1.56, and 1.62 all had similar effects on GUS transcript abundance, consistent with their classification as medium or strong promoters in qualitative activity assays.

### Off‐target effects of dTALE expression in rice

**Figure 8:**
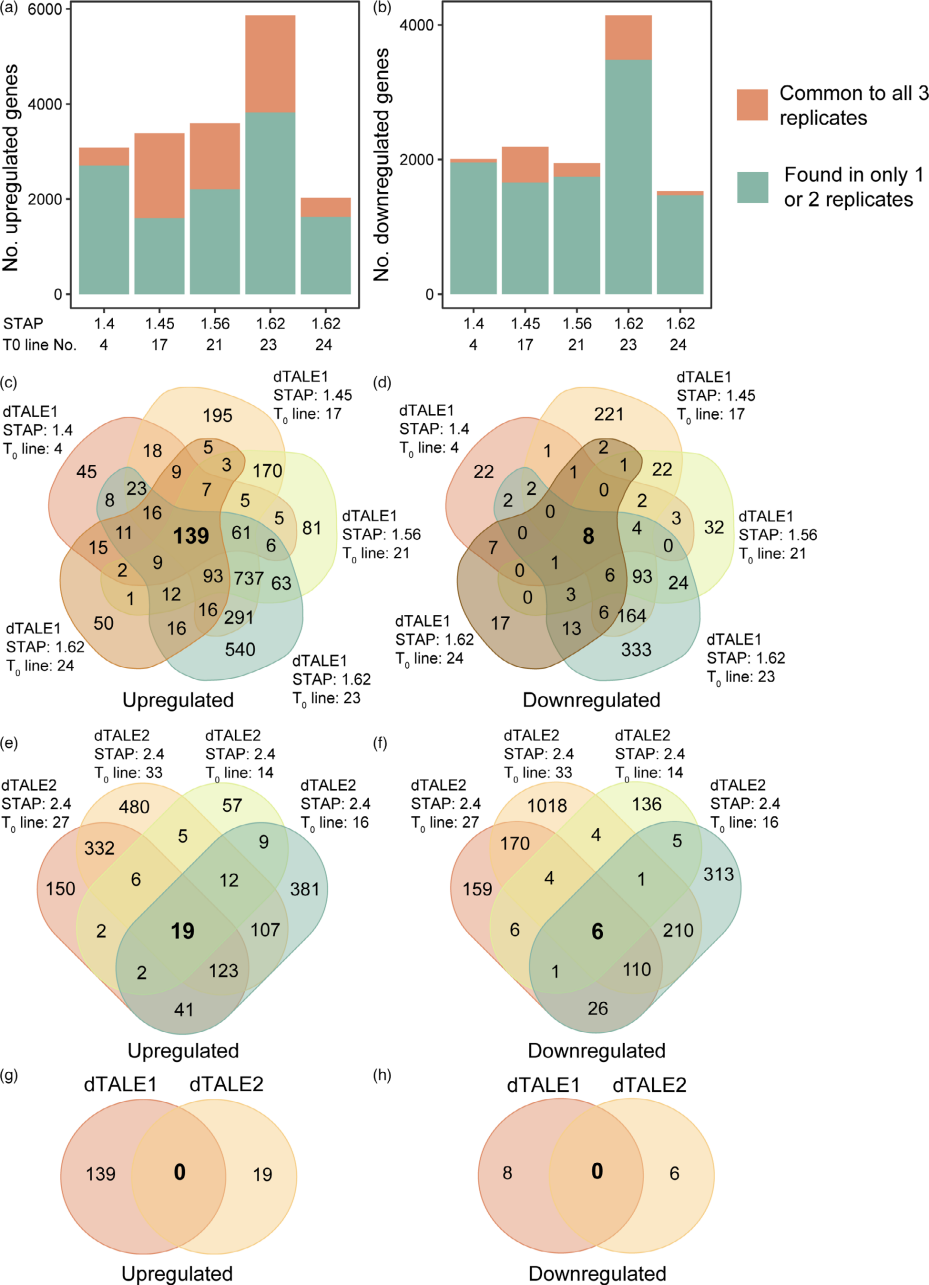
Differentially expressed genes in transgenic lines expressing dTALEs. (a) Number of genes upregulated in *ZjPCK*_*pro*_:dTALE1‐STAP1:GUS lines compared to wild‐type. (b) Number of genes downregulated in *ZjPCK*_*pro*_:dTALE1‐STAP1:GUS lines compared to wild‐type. For both A AND B, each bar depicts the similarity between three different T_2_ lines, descended from three individual homozygous T_1_ seed that were harvested from a single T_0_ plant. The red shaded component of the bar depicts the number of differentially expressed genes that were common to all three T_2_ lines descended from the single T_0_ event. The blue component of the bar depicts the number of differentially expressed genes that were only found in one or two of the respective T_2_ lines. (c) The overlap in upregulated genes between different T_2_ lines shown in a. (d) The overlap in downregulated genes between different T_2_ lines shown in B. e) The overlap in upregulated genes between T_1_ lines harvested from fossur different T_0_ plants expressing either *ZjPCK*_*pro*_:dTALE2‐STAP2.4:GUS or *ZjPEPC321*_*pro*_:dTALE2‐STAP2.4:GUS constructs (3 T_1_ lines per T_0_ event). (f) The overlap in downregulated genes in the lines shown in (e). (g) The overlap in upregulated genes between all transgenic lines expressing dTALE1 and dTALE2. h) The overlap in downregulated genes between all transgenic lines expressing dTALE1 and dTALE2.

Given the inherent role of TALE proteins as transcriptional activators, a differential expression analysis was conducted to determine whether dTALE1 expression in transgenic plants induced ectopic expression of endogenous rice genes. Transcriptomes of 75 T_2_ plants (derived from 15 T_1_ homozygous lines descended from 5 T_0_ *ZjPCK*_*pro*_:dTALE1‐STAP1:GUS plants) were compared to transcriptomes of wild‐type plants to identify genes where transcript abundance was altered (*p*_adj_ < 0.05, fold change >2, Table 3, File S1). Although there were large numbers of differentially expressed genes detected when the pooled T_2_ plants descended from each single T_0_ transgenic event were compared to controls, on average only ~30% of upregulated genes (Figure 8a) and 11% of downregulated genes (Figure 8b) were identified in all 15 biological replicates descended from any T_0_ progenitor line. Notably, comparison of these cohorts of differentially expressed gene sets across all five independent T_0_ progenitor lines revealed just 139 upregulated (Figure 8c, File S2) and 8 downregulated (Figure 8d, File S2) genes in common. Thus, although in any given transgenic line the abundance of a large number of transcripts is altered relative to wild‐type, relatively few of those changes are likely to be directly attributable to the presence of the dTALE1 transgene.

**Table 3:** Number of endogenous rice genes that are up‐ or downregulated in *ZjPCK*_*pro*_:dTALE1‐STAP1:GUS lines as compared to wild‐type (*p*_adj_ < 0.05, fold change >2). Line numbers correspond to those in Figure 7. Gene identities are shown in File S2

STAP #	T_0_ line	T_1_ line	Upregulated	Downregulated
1.4	4	2	2205	1323
1.4	4	5	1761	788
1.4	4	6	716	400
1.45	17	3	2544	1237
1.45	17	5	2564	1203
1.45	17	9	2524	1355
1.56	21	3	1785	552
1.56	21	5	3233	1549
1.56	21	8	1947	557
1.62	23	1	3366	1625
1.62	23	3	3597	1953
1.62	23	8	4636	3247
1.62	24	3	1035	760
1.62	24	6	690	259
1.62	24	8	1657	919

To further assess the likelihood that dTALE1 directly binds to any of the differentially expressed genes, the reference genome of *Oryza sativa ssp. japonica* cv. KitaakeX was searched for potential dTALE1‐binding sites. There are no exact match binding sites or 1 bp mismatch binding sites in the reference genome. However, there are 2 sites in the genome that differ by 2 bp (~90% identity), 36 sites that differ by 3 bp (~85% identity), and 475 that differ by 4 bp (~80% identity, Table 4). Potential off‐target binding sites that differed by 5 bp or more (i.e. <75% identity to the known binding site) were not considered. The position of the potential off‐target binding sites was then evaluated relative to protein‐coding genes, the number of which is reported in Table 5 along with the patterns of differential expression. Only five genes that have potential off‐target binding sites in their vicinity were consistently upregulated in all dTALE1 lines (Tables 5 and 6). Thus, just 3% of the genes that are consistently differentially expressed in the transgenic lines expressing dTALE1 are likely caused by direct proximal binding of dTALE1.

**Table 4:** Number of potential off‐targets of dTALE1 and dTALE2 filtered by number of mismatches

Number of mismatches (off‐targets)	dTALE1	dTALE2
1 bp mismatch sites	0	0
2 bp mismatch sites	2	0
3 bp mismatch sites	36	23
4 bp mismatch sites	475	1361

**Table 5:** Number of genes with a potential 2, 3, or 4 bp‐mismatched dTALE1‐binding site in or near to the gene, categorized by position relative to the gene. Intergenic means within 5 kbp upstream or downstream from the start or stop codon. Number of associated genes that are up or downregulated in all transgenic lines is indicated

Binding site position	# Genes	# Expressed	# Up	# Down
intergenic	283	148	0	0
upstream	136	87	3	0
CDS	98	70	1	0
downstream	58	34	0	0
intron	25	17	1	0
3′ UTR	18	17	0	0
5′ UTR	11	7	0	0

**Table 6:** Identity of the five genes that are consistently upregulated in transgenic lines expressing dTALE1, showing transcripts per million (TPM) detected in each case

Gene ID	Annotation	Off‐target site (mismatches in bold)	Location	dTALE1, TPM	WT, TPM
OsKitaake03g369100	DUF1618	**A**CCCCGCATAGCTG**G**ACA**A**	CDS	1	0
OsKitaake02g392000	DEAD‐Box helicase	TCCCCACATA**TT**TGA**T**CAT	Upstream	39	1
OsKitaake07g118833	Hypothetical	TCCC**A**G**A**ATAG**A**TGAACAT	Upstream	9	5
OsKitaake06g213800	bHLH48	TCCC**A**GCAT**G**GCTG**TC**CAT	Upstream	56	1
OsKitaake04g082200	MAPKKK	T**G**CCCGC**G**T**T**GCTG**G**ACAT	Intron	1	0

Of the five genes with potential off‐target binding sites in their vicinity, only slight changes in abundance were seen for the two with sites either in the coding region or an intron and for one with a site 1.7 kbp upstream of the start codon on the sense strand (Table 6). The other two with sites in the upstream region (OsKitaake02g392000 and OsKitaake06g213800) were more obviously impacted, with 39‐ and 56‐ fold differences in transcript abundance between the dTALE1 transgenic lines and wild‐type, respectively. OsKitaake06g213800 encodes a basic helix–loop–helix transcription factor that is orthologous to both bHLH48 and bHLH60 in *Arabidopsis thaliana* (Figure S6), and OsKitaake02g392000 encodes a DEAD‐box helicase that is orthologous to both AT2G07750 and AT1G63250 in *Arabidopsis thaliana* (Figure S7). In the case of the bHLH gene, the putative dTALE1‐binding site is on the sense strand, one full‐turn of the double helix (11 bp) upstream of the TATA box, which is an optimal position for transcriptional activation. Although the putative binding motif upstream of the DEAD‐Box helicase is also on the sense strand, it is 3.3 kbp upstream of the transcription start site and is positioned in the middle of an intron of an upstream gene, which is itself not differentially expressed in the dTALE1 lines. Of note is that both genes encode proteins that regulate gene expression. As such, it is possible that expression of the remaining genes that are consistently up‐ or downregulated in dTALE1 transgenic lines is altered by these induced regulators as opposed to dTALE1 itself.

To test whether the activation of OsKitaake02g392000 and OsKitaake06g213800 in transgenic lines was most likely due to specific binding of dTALE1 to the identified off‐target sites as opposed to non‐specific transcriptional activation as a result of the presence of a dTALE protein in the nucleus, transcriptomes of lines expressing *ZjPCK*_*pro*_:dTALE2‐STAP2:GUS or *ZmPEPC321*_*pro*_:dTALE2‐STAP2:GUS were analysed (File S1). Analysis of differentially expressed genes in these transgenic lines revealed that, similar to the analysis of dTALE1 transgenic lines, there were few genes that were consistently upregulated (19 genes) (Figure 8e, File S2) or downregulated (6 genes) (Figure 8f, File S2) when dTALE2 expressing plants were compared to control (minus dTALE2) plants. Importantly, there was no overlap between the genes that were consistently upregulated (Figure 8g) or downregulated (Figure 8h) in the dTALE1 and dTALE2 transgenic lines. As with dTALE1, there were no exact matches or sites that differed by 1 bp from the dTALE2‐binding site in the rice genome. There were also no sites that differed by 2 bp and fewer potential off‐target binding sites that differed by 3 bp, but more potential off‐target binding sites differed by 4 bp (Table 4). In total, there are 1080 genes within 5 kbp of the 1384 potential dTALE2 off‐target binding sites of which only 141 are also near genes that were potentially targeted by dTALE1. The position of the potential dTALE2 off‐target binding sites was evaluated relative to protein‐coding genes, the number of which is reported in Table 7 along with the patterns of differential expression. Only two genes that have putative dTALE2‐binding sites in their vicinity were consistently upregulated in all dTALE2 lines (Tables 7 and 8), one encoding a serine–threonine protein kinase and the other an oxidoreductase. In both cases, upregulation was <3‐fold. As such, we conclude that substantial upregulation of the bHLH gene (OsKitaake06g213800) and the DEAD‐Box helicase (OsKitaake02g392000) in dTALE1 lines is attributable to specific properties of dTALE1 as opposed to general effects of dTALE‐mediated transcriptional activation. It should thus be possible to eliminate off‐target gene activation with a truly orthogonal dTALE design.

**Table 7:** Number of genes with a potential 3 or 4 bp‐mismatched dTALE2‐binding site in or near to the gene, categorized by position relative to the gene. Intergenic means within 5 kbp upstream or downstream from the start or stop codon. Number of associated genes that are up or downregulated in all transgenic lines is indicated

Binding site position	# Genes	# Expressed	# Up	# Down
intergenic	845	449	1	0
upstream	113	81	0	0
CDS	27	20	0	0
downstream	80	41	1	0
intron	29	21	0	0
3′ UTR	6	6	0	0
5′ UTR	10	8	0	0

**Table 8:** Identity of the two genes that are consistently upregulated in transgenic lines expressing dTALE2, showing transcripts per million (TPM) detected in each case

Gene ID	Annotation	Off‐target site (mismatches in bold)	Location	dTALE2, TPM	WT, TPM
OsKitaake11g227500	Ser‐Thr protein kinase	TGACG**A**G**A**GATAG**C**TT**T**CA	Downstream	3.4	1.6
OsKitaake08g030300	Oxidoreductase	**A**GACG**T**G**AC**ATAGTTTCCA	Intergenic	5.1	2.2

## Discussion

In a series of experiments in stable transgenic lines, the feasibility of using the dTALE‐STAP system for tissue‐specific multiplexing of transgene expression in rice has been evaluated. In addition to the previously reported dTALE1‐STAP1 system (Brückner *et al.,* 2015), a second version was designed and tested (dTALE2‐STAP). Both dTALE1 and dTALE2 were shown to activate reporter gene expression from their cognate STAPs in stable transgenic rice lines, with strength of expression varying between individual dTALE‐STAP pairings. For any individual STAP, promoter strength also differed in different cell‐types (Figures 2 and 3; Tables 1 and 2). STAPs 1.45/2.45, 1.56/2.56, and 1.62/2.62 drove the strongest bundle sheath cell‐specific expression of GUS, whereas STAPs 1.1/2.1, 1.3/2.3, 1.5/2.5, and 1.62/2.62 were most reliable for strong mesophyll cell‐specific expression. STAP 1.5/2.5, which was one of the strongest in transient expression assays in tobacco (Brückner *et al.,* 2015), was essentially inactive in bundle sheath cells of transgenic rice lines. Collectively these data demonstrate that both dTALE1‐STAP1 and dTALE2‐STAP2 systems can be used to drive gene expression in stable transgenic rice lines, but the activity of any individual STAP sequence must be empirically evaluated in the targeted developmental context.

In addition to variation in STAP strength, the extent to which the two dTALE proteins activated their cognate STAPs was different. Whereas levels of STAP‐driven GUS transcripts in *ZjPCK*_*pro*_:dTALE2‐STAP2:GUS (bundle‐sheath specific) or *ZmPEPC321*_*pro*_:dTALE2‐STAP2:GUS (mesophyll specific) lines were no higher than levels of dTALE2 transcripts in the same lines, significant amplification of GUS transcript levels was observed in corresponding dTALE1 lines (Figure 7). Thus, dTALE activity also needs to be empirically evaluated in stable transgenic lines of the target species. Crucially, however, once dTALE and STAP activities have been determined with single reporter gene constructs in any specific developmental context, relative activities are consistent in multiplexed constructs and those activities are heritable (Figure 5). The majority of variation observed between transgenic lines is manifest at the T_1_ stage. Such variation is common in transgenic rice lines because of the inherent somaclonal variation that occurs when T_0_ plants are regenerated from callus after transformation (Miyao *et al.,* 2011; Wei *et al.,* 2016; Zhang *et al.,* 2014). Thus, with appropriate genotypic and phenotypic validation of T_1_ lines, the dTALE‐STAP system enables transgene expression levels to be varied between different tissues (using weak versus strong dTALEs for different tissues) and/or between different genes in a single tissue (using weak versus strong STAPs for different genes).

As with the introduction of any transcriptional activator, the introduction of dTALE proteins into stable transgenic lines has the potential to unintentionally activate the expression of endogenous genes. Transcriptome analysis of lines harbouring either dTALE1‐ or dTALE2‐containing transgenes revealed a large number of genes that were differentially expressed as compared to control lines (Figure 8). Many of these differences are likely to be associated with the somaclonal variation discussed above. However, in the case of dTALE1, which was not designed specifically for use in rice, greater than 50‐fold upregulation is observed for a gene which has a sequence that differs by only 3 bp from the dTALE1‐binding site, positioned optimally in relation to the TATA box (Grau *et al.,* 2013). Because this off‐target gene encodes a bHLH transcription factor, the Arabidopsis ortholog of which activates transcription of many downstream genes (Yang *et al.,* 2021), significant levels of endogenous gene activation in the presence of dTALE1 are thus predictable. Given that endogenous gene activation will in itself have further downstream effects, downregulation of some genes in the presence of dTALE1 is also expected. The design of dTALE2 took the rice genome sequence into consideration, and fewer genes were consistently upregulated across all dTALE2 containing lines (19 genes) than dTALE1 containing lines (139 genes). These observations suggest that, for any particular species with a known genome sequence, it should be possible to design orthogonal dTALEs that will not activate endogenous gene expression.

Very few attempts to identify orthogonal binding sequences for programmable transcriptional regulators have been reported. One example searched for sequences that differed by at least 3 bp from the dTALE‐binding sequence and were absent from all promoter sequences (up to 2 kbp upstream of the ATG) in the human genome (Garg *et al.,* 2012). However, three mismatches may not be sufficient to prevent binding of the dTALE because there is growing evidence that the impact of mismatches on the ability of RVDs to bind cognate bases depends on the combined effects of RVD‐type, position within the EBE, overall RVD‐composition, and the number of repeats (Juillerat *et al.,* 2015; Meckler *et al.,* 2013; Miller *et al.,* 2015; Rinaldi *et al.,* 2017; Rogers *et al.,* 2015; Streubel *et al.,* 2012). With this in mind, position‐dependent base preferences for canonical RVDs (those with amino acid variants HD, NI, NG, or NN) have been evaluated and have been used to rate the impact of specific RVD‐base mismatches in the context of the repeat array (Erkes *et al.,* 2019; Miller *et al.,* 2015). This allowed putative target sites for natural TALEs to be identified and synthetic dTALEs to be designed (e.g. PrediTALE, Erkes *et al.,* 2019). We used this information to rank potential orthogonal dTALE EBEs and to select the dTALE2 EBE sequence. The activation of fewer endogenous genes in stable rice lines expressing dTALE2 than in lines expressing dTALE1 suggests that our approach was successful. However, dTALE2 is an inherently weak transcriptional activator *in planta* and thus our design strategy for orthogonal dTALEs needs to be further improved and validated using versions with strong activation potential. Further optimization should also take into account the exact composition of strong versus intermediate/weak RVDs because the presence of multiple strong RVDs (e.g. HD for cytosine and NN for guanine) can compensate for mismatches, particularly in longer repeat arrays (Rinaldi *et al.,* 2017; Rogers *et al.,* 2015; Streubel *et al.,* 2017; Wicky *et al.,* 2013). Together, these factors suggest that complete orthogonality could be achieved if fewer RVD repeats and a reduced number of strong RVDs were incorporated into the central repeat domain of the dTALE.

Although a number of systems for the manipulation of transgene expression in plants have been reported, most have shortcomings. For example, artificial transcription factors that are based on fixed DNA‐binding domains have been used for tissue‐specific and multiplexed regulation of plant transgene expression but heritability was not investigated (Belcher *et al.,* 2020; Brophy *et al.,* 2022). Furthermore, off‐target activation of endogenous genes was not considered, and if proven to be a problem, fixed DNA‐binding domains cannot be adapted to prevent such activation. Programmable DNA‐binding domains such as zinc finger domains (ZF), TALE repeat domains, and CRISPR‐derived RNA‐guided DNA‐binding domains provide an appropriate alternative, because DNA‐binding specificity can be adapted and designed to be orthogonal. Of these, ZF‐based DNA‐binding domains are not truly modular and are not easy to design (Voytas and Gao, 2014), leaving only TALEs and CRISPR‐based systems as viable options for programmable specific DNA binding within synthetic circuits.

The simultaneous regulation of several genes is possible with both TALE‐ and CRISPR‐based systems, with CRISPR‐based activator systems having the advantage that multiplexing can be achieved using either multiple sgRNAs or sgRNA arrays. RNA Polymerase I and RNA Polymerase II‐driven sgRNA arrays can be processed by t‐RNAs, ribozymes, Csy4 target sequences or even by endogenous plant RNAses (Čermák *et al.,* 2017; Uranga *et al.,* 2021; Xie *et al.,* 2015). Although it is currently unclear how many individual gRNAs can be processed using CRISPR‐based multiplex strategies, this approach has the potential to facilitate direct activation of multiple endogenous target genes with a single Cas activator protein (e.g. dCas9‐TV (Li *et al.,* 2017) or dCas12a‐TV (Ming *et al.,* 2020)). By contrast, with dTALEs, one individual dTALE would be required for each endogenous target gene. That said, the need for multiple guide RNAs increases the risk of activating off‐target gene expression, a situation that is minimized in the dTALE‐STAP system by having only one target EBE.

The design of orthogonal RNA‐guides for DNA binding in CRISPR systems is constrained because strong binding of Cas9 is achieved even if only 8 of the 20 PAM proximal nucleotides (as well as the PAM) are identical (Singh *et al.,* 2016). As such, putative orthogonal targets for dCas9‐based transcription factors have a maximum size of 11 base pairs, making it very challenging to identify such sequences in species with large genomes. dCas12a‐based systems may provide an appropriate alternative because DNA binding is only accomplished if 17 of the 20 PAM proximal nucleotides are identical (Jeon *et al.,* 2018). This would increase the length of an orthogonal Cas12a target to 21 nucleotides. However, this advantage is offset by the fact that the strength of the dCas12‐TV is only moderate (Ming *et al.,* 2020). Recently the potency of CAS activators has been increased by engineering CRISPR‐Act2.0 (Lowder *et al.,* 2018) and CRISPR‐Act3.0 (Pan *et al.,* 2021). However, these engineered versions come with additional components and the activation domains are decoupled from the dCas proteins, introducing the possibility of unpredictable off‐target effects. Given information from the study of natural TALEs, orthogonal design may prove to be more predictable. For example, the natural TALE AvrBs3, which induces expression of the Bs3 allele, does not activate the related Bs3‐E allele even though 11 bp of the putative EBE‐binding site are identical to the sequence in the induced allele (Römer *et al.,* 2009). Considering all of these factors, the possibility of identifying orthogonal target sequences that will be activated by a strong transcriptional activator are currently higher with a dTALE than with CRISPR‐based systems.

Thus far, multiple endogenous genes have been constitutively activated in stable transgenic plants using both CRISPR‐based and engineered TALE activators, but tissue‐specific expression has not been reported (Lowder *et al.,* 2018; Morbitzer *et al.,* 2010; Pan *et al.,* 2021; Xiong *et al.,* 2021). However, inducible and constitutive knockouts using tissue‐specific promoters to drive Cas9 (or estradiol‐inducible transcription factors) have been reported and in principle similar approaches could be used for tissue specific activation of multiple endogenous genes (Decaestecker *et al.,* 2019; Feder *et al.,* 2020; Schindele *et al.,* 2022; Wang *et al.,* 2020). An alternative would be to use either endogenous or synthetic transcription factors to induce orthogonal synthetic promoter sequences that are not present in the host genome, similar to the approach we used here for dTALEs in rice. Such sequences have been tested in stable transgenic lines of dicotyledonous species but tissue‐specific expression has not yet been reported (Cai *et al.,* 2020). Whilst recognizing that further optimization is required, we conclude that the dTALE‐STAP system currently provides a powerful tool for regulating and fine‐tuning the expression of multiple transgenes in different spatial and/or temporal contexts during plant development.

## Methods

### Plant materials and growth conditions

All experiments were carried out using *Oryza sativa spp. japonica* cv. Kitaake. Unless otherwise indicated, plants were grown in a controlled environment chamber (Model PGC Flex, Conviron, Winnipeg, MB, Canada) with a 16 h light/8 h dark photoperiod, light intensity of 400 μmol photons/m^2^/s, temperatures of 28 °C and 22 °C during the day and night, respectively, and 60% relative humidity. Irradiance was supplied by a mixture of fluorescent tubes (Master TL5 HO 54W/840, Philips Lighting, The Netherlands) and halogen incandescent globes (42 W 2800 K warm white clear glass 630 lumens, CLA, Brookvale, Australia). Plants were individually grown in 1 L pots in a soil mix composed of 80% peat/10% perlite/10% vermiculite (pH 5.6–5.8) mixed with 5 g of slow‐release fertilizer (Osmocote, Evergreen Garden Care, Australia) supplied once at the beginning of the growth cycle. All pots were kept at field water capacity.

### Construct assembly

dTALE1 and ten STAP1 clones from Brückner *et al*. (2015) were obtained as Golden Gate compatible Level 0 modules (Engler *et al.,* 2014; Weber *et al.,* 2011). Constructs to test individual STAP1s were assembled from Level 1 modules in which the *hpt* gene is driven by the rice *Actin1* promoter (*OsAct1*_*pro*_) (McElroy *et al.,* 1990), dTALE1 is driven by the *Zoysia japonica PHOSPHOENOLPYRUVATE CARBOXYKINASE* (*ZjPCK)* promoter (*ZjPCK*_*pro*_) (Nomura *et al.,* 2005) and the ten STAP1s (1.1, 1.3, 1.4, 1.5, 1.7, 1.13, 1.21, 1.45, 1.56, or 1.62) drive expression of the β‐glucuronidase (GUS) coding sequence (no introns included). All transcriptional units were terminated by the *nos* terminator. Level 2 constructs were assembled into the binary vector pAGM4723. For constructs containing multiple STAP1s, STAPs 1.1, 1.3, 1.4, and 1.5 were assembled into Level 1 modules with GUS, maize NADP‐dependent malic enzyme (*Zm*ME), eGFP, and kOrange coding sequences, respectively. Fluorescent reporter proteins (eGFP and kOrange) were linked to a nuclear localization signal‐encoding sequence (NLS; Luginbuehl *et al.,* 2020). All Level 1 modules contained the *nos* terminator. Level 2 constructs contained the *hpt* gene driven by *OsAct1*_*pro*_ and were assembled into the binary vector pAGM4723. Two different Level 2 constructs were tested, one with *ZjPCK*_*pro*_ driving expression of dTALE1 and a second with *ZjPCK*_*pro*_ driving expression of the dsRed fluorescent protein as a negative control.

Level 2 constructs to test individual STAP2s with dTALE2 contained the *hpt* gene driven by *OsAct1*_*pro*_, dTALE2 STAP2s (STAPs 2.1, 2.3, 2.4,2.5, 2.13, 2.21, and 2.62) upstream of the GUS coding sequence (intron‐less) and dTALE2 expressed either from *PHOSPHENOLPYRUVATE CARBOXYLASE (PEPC)* promoters from *Zea mays* (*ZmPEPC321*_pro_) (Gupta *et al.,* 2020; Matsuoka *et al.,* 1994) or *Setaria viridis* (*SvPEPC500*_pro_) (Gupta *et al.,* 2020), or from *ZjPCK*_*pro*_. A negative control version of the *ZmPEPC321*_*pr*o_ construct was also generated with mTurquoise fluorescent protein in place of dTALE2. STAP2s compatible with dTALE2 (containing the dTALE2‐EBE) were generated via PCR as Golden Gate modules in which dTALE1 EBE was exchanged for the dTALE2 EBE. For constructs containing multiple STAP2s, STAPs 2.1, 2.3, 2.4, and 2.5 were assembled into Level 1 modules with maize *PHOSPHOENOLPYRUVATE DIKINASE* (*ZmPPDK*), *PEPC* (*ZmPEPC*), *MALATE DEHYDROGENASE* (*ZmMDH*) and *CARBONIC ANHYDRASE* 1 (*ZmCA1*) coding sequences, respectively. *Zm*CA1 was linked to an AcV5 tag to assist with protein detection (Lawrence *et al.,* 2003). Two different Level 2 constructs were generated, one driven by *ZmPEPC321*_pro_ and the other driven by *SvPEPC500*_pro_.

All dTALE and STAP sequences are shown in Figure S8.

### Plant transformation

For *ZjPCK*_*pro*_:dTALE1:STAP1 and direct *ZjPCK*_*pro*_:GUS constructs, calli were obtained from sterilized dehulled seeds and incubated with *Agrobacterium tumefaciens* strain AGL1 or EHA105. Callus induction, regeneration and selection of positive seedlings was performed as described in a modified protocol of Toki *et al*. (2006) that can be downloaded from https://langdalelab.files.wordpress.com/2018/06/kitaake‐rice‐transformation.pdf. Transgenic plants were isolated based on their resistance to hygromycin. After formation of roots, T_0_ plants were moved to soil and grown to seed in the controlled environment chamber as above (*ZjPCK*_*pro*_:dTALE1:STAP1) or at 30 °C 16 h 300 μmol photons/m^2^/s light/25 °C 8 h dark photoperiod (*ZjPCK*_*pro*_:GUS).

For dTALE2‐STAP2 constructs, calli were obtained from sterilized dehulled seeds and incubated with *A. tumefaciens* strain AGL1, EHA105, or LBA4404. Callus induction was carried out on NB medium (4 g/L of N6 salts and vitamins, 30 g/L of sucrose, 0.1 g/L of myo‐inositol, 1 g/L of casamino acid, 2.8 g/L of L‐proline, 3 g/L of phytagel, pH 5.7) with 2 mg/L of 2,4‐D for 1 month at 32 °C with a 500 μmol of photons/m^2^/s 16 h light/8 h dark cycle (subcultured to fresh medium at 2 weeks). Actively growing calli were transferred to NB‐AS medium (NB plus 100 μM acetosyringone, 1% glucose, pH 5.2) for 3 days at 25 °C with a 16 h light/8 h dark cycle, before co‐culture with *A. tumefaciens*. Growth of *A. tumefaciens* was subsequently suppressed by incubation in MS liquid medium supplemented with 250 mg/L of cefotaxime for five periods of 1 h at 25 °C, washing repeatedly with sterile water in between incubations, followed by overnight incubation in MS liquid medium supplemented with 500 mg/L of cefotaxime (25 °C with a 16 h light/8 h dark cycle). Washing and overnight incubation in cefotaxime was repeated for 3 consecutive days. Callus selection was carried out on NB medium supplemented with 200 mg/L of timentin and 30 mg/L of hygromycin for 3 weeks at 28 °C (16 h light/8 h dark cycle) before the transfer to PM medium (4 g/L of N6 salts and vitamins, 0.3 g/L of casein, 0.1 g of myo‐inositol, 0.5 g/L of glutamine, 90 g/L of sorbitol, 0.5 mg/L of 2′4‐D, 1 mg/L of 1‐naphthaleneacetic acid (NAA), 0.5 mg/L of 6‐benzylaminopurine (BA), 3 g/L of phytagel, pH 5.7) supplemented with 200 mg/L of timentin for 1 week at 28 °C (16 h light/8 h dark cycle). Calli were regenerated on RM medium (4 g/L of N6 salts and vitamins, 0.3 g/L of casein, 0.1 g of myo‐inositol, 0.5 mg/L of NAA, 5 mg/L of kinetin, 30 g/L of sucrose, 5 g/L of glucose, 3 g/L of phytagel, pH 5.7) supplemented with 200 mg/L of timentin and 25 mg/L of hygromycin for 4 weeks at 25 °C (16 h light/8 h dark cycle), sub‐cultured to fresh medium at 2 weeks. Shooting plants were transferred to glass jars containing solid MS medium supplemented with 50 mg/L of hygromycin for 2–3 weeks at 25 °C (16 h light/8 h dark cycle) and were then acclimated in water for 2–3 days before transferring to 1/4 Kimura solution for another 5–6 days at 28 °C with a 16 h light/8 h dark cycle. Acclimated transgenic plants were either grown in the greenhouse (December to February) or transplanted to the open GM‐field at National Chung‐Hsing University (March to November).

Transgene copy number was estimated in regenerating T_0_ seedlings through quantification of hygromycin phosphotransferase (*hpt*) gene copy number by digital droplet PCR (ddPCR) (at either IDna Genetics Ltd. Norwich, UK or Institute of Molecular Biology Genomics Core at Academia Sinica (QX100 Droplet Digital PCR System, BIO‐RAD).

### Histochemical detection of GUS activity

GUS staining of dTALE1 lines was performed using the mid‐distal region of the youngest fully expanded leaf of 4‐week‐old plants. Using a razor blade, thin transverse leaf sections and 1‐mm leaf strips were obtained from fresh tissue and placed into staining solution (1 mm 5‐bromo‐4‐chloro‐3‐indolyl ß‐D‐glucuronide (X‐Gluc) in 0.1 m sodium phosphate buffer, pH 7.0, 10 mm EDTA, 0.1% Triton X‐100, 0.4 mm of potassium ferricyanide and 0.4 mm of potassium ferrocyanide), subjected to vacuum infiltration for 15 min releasing the vacuum every 2 min, and then incubated at 37 °C for at least 30 min or until blue coloration developed. Chlorophyll was removed by washing the sections and strips with 70% ethanol thrice, 1 h each wash, then with 90% ethanol overnight. Cleared leaf sections and strips were incubated in 50% glycerol for 1 h prior to mounting onto a glass slide. Imaging was performed using a Leica DM5500 automated upright microscope (Leica Microsystems, Wetzlar, Germany) under a 20x objective and brightfield channel.

GUS staining of direct *ZjPCK*_*pro*_:GUS lines, and of the *ZjPCK*_*pro*_:dTALE1‐multi STAP1 line, was performed using the middle section of fourth fully expanded leaves from plants that had been grown in a mixture of 1:1 topsoil and sand for 2 weeks in a controlled environment growth room (28 °C day/25 °C night with a photoperiod of 12 h of light and 12 h of dark, relative humidity of 60% and light intensity of 400 μmol photons/m^2^/s). Leaf tissue was fixed in 90% (v/v) acetone at 4 °C overnight and rinsed with phosphate buffer (pH 7.0) prior to staining and clearing as for dTALE1 lines. Cross sections were prepared manually using a razor blade prior to imaging using an Olympus BX41.

GUS staining of dTALE2 lines was performed using the youngest fully expanded leaves from T_0_ plants, 3 weeks after removal from tissue culture medium. Fresh sections were sampled using a Leica VT1200 Microtome (Leica Microsystems GmbH) and GUS staining was carried out as for dTALE1 lines. Chlorophyll was removed by incubating in 70% ethanol at 65 °C for 1 h and then samples were imaged using a Nikon upright microscope (ECLIPSE Ni‐U, Nikon Co.).

### Quantitative RT‐PCR

Leaf discs were collected from the mid‐distal leaf blade portion of the youngest fully expanded leaf from the central shoot of 4‐week‐old rice plants, frozen in liquid N_2_ and stored at minus 80 °C. Frozen samples were homogenized using a Qiagen TissueLyser II (Qiagen, Venlo, The Netherlands). RNA was extracted using an RNeasy Plant Mini Kit (Qiagen, Venlo, The Netherlands). DNA from the samples was removed using an Ambion TURBO DNA free kit (Thermo Fisher Scientific, Tewksbury, MA) and RNA quality was determined using a NanoDrop (Thermo Fisher Scientific, Tewksbury, MA). One microgram of RNA was reverse transcribed into cDNA using SuperScript™ III Reverse Transcriptase (Thermo Fisher Scientific, Tewksbury, MA). For multigene dTALE1‐STAP1 lines, qPCR and melt curve analyses were performed on a Viia7 Real‐time PCR system (Thermo Fisher Scientific, Tewksbury, MA) using the Power SYBR green PCR Master Mix (Thermo Fisher Scientific, Tewksbury, MA) according to the manufacturer's instructions. Primer pairs were designed using Primer3 in Geneious R9.1.1 (https://www.geneious.com):

5′‐CCGACATGTGGAGTGAAGAG & 5′‐GCAAAATCGGCGAAATTCCA (GUS);
5′‐CCGGCAGTACTATCTCAGGA & 5′‐CTCTCGTCCGTTTCCTGAAG (*Zm*ME);
5′‐CGCACCATCTTCTTCAAGGA & 5′‐ACGTTGTGGCTGTTGTAGTT (eGFP);
5′‐CTGCCGATGGTCCTATCATG & 5′‐TCTTTTGCCGCCTTGTAAGT (kOrange);
5′‐CTGTCAACTGCCGCAAGAAG & 5′‐GGCGAGTGACGCTCTAGTTC (Ubiquitin).

For multigene dTALE2‐STAP2 lines, RT‐PCR was performed as above using the primers listed in Ermakova *et al*. (2020):

5′‐GAATCCCAGAGCATCCCGAG & 5′‐GTGCAGGACAGGGAAACGTA (*Zm*PPDK);
5′‐CGTCATACAAGCCGGCAATG & 5′‐TGTGCTTCCAGACTCTGCAG (*Zm*PEPC);
5′‐GCCCCTCTCGGCCGC & 5′‐CACCTTCGAGGGCTTGAAACG (*Zm*MDH);
5′‐AGGTTCTCCAGGGACAGGTT & 5′‐GGACGGGTTCCACAAGTTCA (*Zm*CA);
5′‐TGCCGTGCTCATCATTGACT & 5′‐TTGTCAGGGTTGTAGCCGAC (elongation factor 1 alpha).

Relative fold change was calculated by the 2^−ΔΔCt^ method, using the geometric mean of the reference gene (Ubiquitin) Ct values (Livak and Schmittgen, 2001). Amplicons were visualized on 1% agarose gels.

### Protein detection on blots

Leaf discs of about 0.4 cm^2^ were collected from the mid‐distal region of the youngest fully expanded leaves of 4‐week‐old plants, immediately frozen in liquid N_2_ and kept at minus 80 °C. Leaf discs were homogenized in ice‐cold extraction buffer containing 50 mm 4‐(2‐Hydroxyethyl)‐1‐piperazinepropanesulphonic acid‐NaOH pH 7.8, 5 mm of MgCl_2_, 2 mm of ethylenediaminetetraacetic acid (EDTA), 5 mm of dithiothreitol, 1% (v/v) polyvinylpolypyrrolidone, 0.1% (v/v) Triton X‐100, and 1% (v/v) protease inhibitor cocktail (P9599; Sigma, St Louis, Missouri, US). Protein extracts were supplemented with 2% (w/v) sodium dodecyl sulphate and incubated at 65 °C for 10 min before being separated by SDS‐PAGE, blotted and probed with specific antibodies against *Zm*ME (1:5000 dilution; Sonawane *et al.,* 2018),GFP (SAB4301138; Sigma, St. Louis, Missouri, US; 1:10 000 dilution), *Zm*PPDK (Langdale lab, University of Oxford, UK; 1:20 000 dilution), *Zm*PEPC (1:10 000 dilution; Karki *et al.,* 2020), ZmMDH (Langdale lab, University of Oxford, UK; 1:5000 dilution), and AcV5 tag (ab49581, Abcam, Cambridge, UK; 1:10 000 dilution) as in Ermakova *et al*. (2019).

### Protein detection by immunolocalization

For fluorescent immunodetection of proteins, leaf tissue was cut directly into fixing solution (4% paraformaldehyde, 0.2% glutaraldehyde, 0.01% Tween‐20, 25 mm sodium phosphate buffer, pH 7.2) and vacuum‐infiltrated until the tissue sank. Tissues were transferred into fresh fixative solution and incubated for 3–4 h at 4 °C. After rinsing in 25 mm sodium phosphate buffer, thin leaf sections were hand‐cut using a razor blade and placed into blocking solution (20 mm of 2‐amino‐2‐(hydroxymethyl)‐1,3‐propanediol, 154 mm of NaCl, 0.1% Tween 20, 3% dried milk powder). Sections were incubated overnight with primary antibody in blocking solution: 1:100 dilution for *Zm*PPDK (Langdale lab, University of Oxford, UK); 1:1000 dilution for *Zm*PEPC (Karki *et al.,* 2020); 1:100 dilution for *Zm*MDH (Langdale lab, University of Oxford) and 1:100 dilution for AcV5 tag (ab49581, Abcam, Cambridge, UK). For visualization, sections were incubated with 1:200 dilution AlexaFluor 488‐conjugated goat anti‐rabbit antibody (A‐11070; Thermo Fisher Scientific, Waltham, Massachusetts, US) (for AcV5: 1:200 dilution AlexaFluor 488‐conjugated goat anti‐mouse antibody; ab150117, Abcam, Cambridge, UK) for 2 h in the dark and treated for 5 min with 0.05% calcofluor white to stain cell walls. Sections were examined with an LSM780 UV‐NLO (Zeiss, Oberkochen, Germany) confocal microscope and fluorescence signal was collected at 546–600 nm for Alexa Fluor 488 (excitation 488 nm), 434–445 nm for cell walls (excitation 405 nm) and 650–742 nm for chlorophyll autofluorescence (excitation 633 nm). For localization of GFP signal, thin leaf sections from fresh leaf tissue were used and fluorescence signal was collected at 499–511 nm emission wavelength after excitation with a 488 nm laser. Images were processed using ImageJ software (National Institutes of Health).

### RNA‐Seq sample collection and pooling strategy

To minimize biological variation due to sampling error, three biological replicates of each *ZjPCK*_*pro*_:dTALE1‐STAP1:GUS transgenic event were grown. T_2_ seed derived from three homozygous T_1_ lines for each independent insertion event were used—that is, STAP1.4—lines 4.2, 4.5, 4.6; STAP1.45 —lines 17.3, 17.5, 17.9; STAP1.56—lines 21.3, 21.5, 21.8; and STAP1.62—lines 23.1, 23.3, 23.8, and lines 24.3, 24.6, 24.8. Seed were sterilized and germinated on MS‐hyg media (2.15 g/L of MS salts and vitamins, 15 g/L sucrose, 0.5 g/L of 2‐(N‐morpholino) ethanesulphonic acid (MES) pH 5.8, 2 g/L phytagel, 20 mg/L hygromycin) in a growth cabinet with a 16 h light/8 h dark photoperiod, light intensity of 150 μmol photons/m^2^/s, 24 °C constant temperature and relative humidity of 60%. After 7 days, plants were transferred into 0.5 L pots with soil and grown in the controlled environment chamber for a further 27 days until the 4th leaf was fully expanded. Leaf tissue (0.5 cm^2^) from the mid‐distal leaf blade portion of the 4th fully expanded leaf was collected from ten plants per line, pooled into a 2 mL centrifuge tube, frozen in liquid N_2,_ and stored at minus 80 °C. For each line, five technical replicate pools were obtained. Samples were collected between 9 am and 11 am on the same day. Frozen samples were homogenized using a TissueLyser II and RNA was extracted using an RNeasy Plant Mini Kit. DNA was removed from the samples using an Ambion TURBO DNA free kit and RNA quality was determined using a NanoDrop.

For dTALE2‐STAP2:GUS lines, T_1_ seeds were sterilized and germinated in soil at 28 °C with 16 h light (400 μmol photons/m^2^/s) and 8 h dark cycle. Two independent T_1_ lines each of *ZjPCK*_*pro*_:dTALE2‐STAP2.4:GUS and *ZmPEPC*_*pro*_:dTALE2‐STAP2.4:GUS were grown, plus one T_1_ line of *ZmPEPC*_*pro*_:mTurquoise‐STAP2.4:GUS. DNA was extracted from leaf 2 of 21‐day old seedlings for genotyping by PCR, using HptF: CGCAAGGAATCGGTCAATACA & HptR: GATGCCTCCGCTCGAAGTAG primers to amplify the *hpt* transgene sequence. Plants with confirmed transgene insertions were selected for RNAseq analysis. For each T_1_ line, leaf 4 was collected from three individual 21‐day old seedlings and RNA was extracted using Trizol® reagent (Invitrogen, Waltham, Massachusetts, US) and the RNeasy Plant Mini Kit. The quality of total RNA was checked using an Agilent Technologies 2100 Bioanalyzer (Agilent Technologies, Santa Clara, California, US) by the Institute of Molecular Biology Genomics Core at Academia Sinica.

RNA was submitted to BGI Tech Solutions Co., Limited (Tai Po, N.T., Hong Kong) for library construction and DNBSEQ eukaryotic strand‐specific transcriptome resequencing at paired‐end 100 bp read length with 30 million clean reads per sample. All RNA‐Seq samples used in this study are available to download from EBI array express under dataset accession number E‐MTAB‐11446.

### Quantification of transcripts and identification of differentially expressed genes

The *Oryza sativa ssp. japonica* cv. KitaakeX 499 v3.0 genome and associated v3.1 annotation files were downloaded from Phytozome (Goodstein *et al.,* 2012). To enable quantification of transgenes in transgenic lines, the transcript file was amended to include the coding sequence (with UTR sequences included) of all genes in the constructs. This transcript file was indexed with Salmon v1.4.0. Paired end read files were then mapped to this index using Salmon v1.4.0 (Patro *et al.,* 2017) with the library type set to the appropriate setting for the library construction method used for RNA‐Seq (‘ISR’). All TPM values for expressed genes are shown in File S3. Differentially expressed genes were identified using DESeq2 (Love *et al.,* 2014). For the purposes of this analysis, only genes with an adjusted *P*‐value < 0.05 and a fold change >2 were considered to be differentially expressed.

### Identification of putative off‐target sites for dTALE1

The sequences of the dTALE1‐ and dTALE2‐binding sites were searched against the *Oryza sativa ssp. Japonica cv*. Kitaake X genome using the glistquery algorithm from the GenomeTester4 toolkit (Kaplinski *et al.,* 2015) while varying the mismatch parameter. The positions of these potential binding sites in the reference genome were identified using blastn setting the percent identity to 100% and word size to 19. The genes within a maximum 5 kbp distance to each potential binding site were recorded and the position of the potential off‐target binding sites analysed.

### Design of rice orthogonal EBEs

The EBE of dTALE2‐specific STAPs was designed to be orthogonal to the rice genome. As a first step, 19 bp‐long putative dTALE DNA‐binding sites (19mers; effector‐binding element—EBE) were randomly generated with the KeeSeeK algorithm (Falda *et al.,* 2014) using optimized parameters with a minimal edit distance of three (parameters: ‐N ‐n 3 ‐a 4:5:5:5 ‐R 0 ‐d 3 ‐K 10 000 ‐t 10 000 ‐v 1 ‐k 2) (Figure S1A). All 19mers with a T at position 0 (T_0_, essential for dTALE binding) were selected and aligned to the rice genome (*Oryza sativa ssp. japonica* variety Nipponbare—accessed through ENSMBL plants via accession number GCA_001433935.1) using glsearch36, with adapted parameters to avoid gaps in the alignments (parameters: ‐b = 200 ‐n ‐T 8 ‐f − 100 ‐m 8CB). Many 19mers (putative TALE‐EBEs) were identified that had several off‐targets in the rice genome containing 3 mismatches (no 1 bp or 2 bp mismatch off‐targets were identified). Importantly, mismatches can have different impacts on dTALE‐DNA interactions and it is possible that an off‐target with 3 or even more mismatches could be bound and induced by a corresponding dTALE. The impact of a mismatch on dTALE‐DNA interaction depends on the kind of the RVD‐base mismatch and the position of the RVD‐base mismatch within the corresponding EBE (Erkes *et al.,* 2019; Meckler *et al.,* 2013; Miller *et al.,* 2015; Rogers *et al.,* 2015). Generally, T_0_‐proximal mismatches are less tolerated then T_0_‐distal mismatches. In order to rate the orthogonality of a given 19mer, a scoring system was implemented using R (version 3.2.0) that was based on position‐dependent average RVD‐base preference (identified based on DNA binding (Miller *et al.,* 2015) and transcriptional activation (Erkes *et al.,* 2019); Figure S1). For the 200 closest off‐targets with 3 bp and more mismatches in the 19mer, an off‐target score was calculated. The off‐target score (S_OFF_) was calculated as the sum of individual mismatch scores (S_M_) multiplied by the position factor (F_P_) (Figure S1B). The mismatch score rates the kind of mismatch and the position factor weights the S_M_ for mismatches that are T_0_ proximal over mismatches that are T_0_ distal.

$$F_{P}=1/\left(\text{position}\;{\text{of mismatch}}^{0.5}\right)$$

$$S_{M}={\left(\text{average}\;\mathrm{RVD}-\text{base preference}–0.5\right)}^{*}-2$$

$$S_{\mathrm{OFF}}=\;\sum \left({F_{P}}^{*}\;S_{M}\right)$$

Off‐target scores (S_OFF_) were calculated for each of the 200 closest off‐targets for each individual 19mer. Each 19mer therefore had 200 off‐target scores (S_OFF1_ → S_OFF200_). The average of the 200 off‐target scores was described as the ‘neverword’ score (S_N_). dTALEs that target a 19mer with a high S_N_ score are more likely to be orthogonal to the rice genome than dTALEs that target 19mers with a lower S_N_.

$$S_{N}=\sum \left(S_{\text{OFF1}}\to S_{\mathrm{OFF}200}\right)/200$$

Notably, S_N_ does not give any information about the distribution of individual off‐target scores in the group of 200 off‐targets and as such the same S_N_ can represent either some very high off‐target scores that are compensated for by some very low off‐target scores, or alternatively a number of equally distributed intermediate off‐target scores. An equal distribution of individual off‐target scores is preferable to ensure dTALE orthogonality against all potential off‐targets. To rate the distribution of the 200 individual off‐target scores for each 19mer, the ratio between the average of the 100 lowest off‐target scores and the average of the remaining 100 scores was calculated. The closer the ratio is to 1, the more equally distributed the individual off‐target scores. The EBE of dTALE2 was thus designed using the 19mer with the highest S_N_ score and the ratio closest to 1 (Figure S1C).

## Conflicts of interest

The authors have no conflicts of interest to declare.

## Author contributions

FD and SFL carried out analysis of single STAP:GUS lines and prepared RNA for RNA‐seq; TS and BA designed the scoring system for orthogonal EBE identification in the dTALE2 system; ME, DV, JLF, and ASH assembled constructs for transformation; YSC generated dTALE2 transformants; LH, ME, and FD analysed multi‐STAP lines; SK designed the transcriptome experiments and carried out bioinformatic analyses; SvC and RF supervised FD and ME; AT supervised TS and BA; SMY supervised SFL and YSC; JMH supervised LH; JAL conceived the study and supervised DV, JLF, and ASH; FD, TS, and JAL wrote the first draft of the manuscript; all authors contributed to the final version of the manuscript.

## Supporting information


**File S1** Gene IDs of upregulated and downregulated genes in individual dTALE1 and dTALE2 transgenic lines.

**File S2** Gene IDs of upregulated and downregulated genes in all dTALE1 and all dTALE2 transgenic lines.

**File S3** Transcript per million (TPM) values for all genes in transcriptome datasets.

**Figure S1** dTALE scoring system and RVD‐composition of rice orthogonal dTALE2.
**Figure S2** β‐glucuronidase (GUS) staining in *ZjPCK*_*pro*_:dsRed‐STAP1:GUS rice transformants.
**Figure S3** β‐glucuronidase (GUS) activity in *ZmPEPC321*_*pro*_:mTurquoise‐STAP2:GUS rice transformants.
**Figure S4** Quantification of bundle sheath and mesophyll cell area.
**Figure S5** Linear regression and ANOVA analyses to determine what proportion of variance in GUS transcript abundance in *ZjPCK*_*pro*_:dTALE1‐STAP1‐GUS lines was attributable to different factors.
**Figure S6** Maximum likelihood phylogenetic tree of the orthogroup containing OsKitaake06g213800.
**Figure S7** Maximum likelihood phylogenetic tree of the orthogroup containing OsKitaake02g392000.
**Figure S8** dTALE and STAP sequences.
