# Supplementary material for: A single promoter‐TALE system for tissue‐specific and tuneable expression of multiple genes in rice
Source: Plant Biotechnol J. 2022 Jun 24;20(9):1786–806. doi: 10.1111/pbi.13864 (PMC9398400; doi:10.1111/pbi.13864)

**SUPPORTING INFORMATION**

**SUPPORTING FILES**

**Supporting File 1.** Gene IDs of upregulated and downregulated genes in individual dTALE1 and dTALE2 transgenic lines.

**Supporting File 2.** Gene IDs of upregulated and downregulated genes in all dTALE1 and all dTALE2 transgenic lines.

**Supporting File 3.** Transcript per million (TPM) values for all genes in transcriptome datasets.

**SUPPORTING FIGURES**

**Supporting Figure 1. dTALE scoring system and RVD-composition of rice orthogonal dTALE2. A)** 19 bp long putative dTALE-binding sites were generated randomly and aligned to the rice genome with the goal of identifying 19mers that are most distant from the rice genome. For every 19mer the 200 closest off-targets were used to rate orthogonality (defined by the S_N_ score). **B)** Average RVD1-dependent base-preferences for position 0 and average RVD-base-preferences for positions 1 to 18 were used for calculation of the mismatch score (S_M_). To weight T_0_-proximal mismatches over T_0_-distal mismatches each mismatch score S_M_ was multiplied with the position factor (F_P_). One exception was the F_p_ for position zero. To weight T_0_ mismatches over all other mismatches the position factor for position zero was defined as 2. The off-target score for each off-target was calculated as the sum of the individual position-dependent mismatch scores. The S_N_ score is the average of the off-target scores of the 200 closest off-targets. **C)** Orthogonal dTALE2 RVD-composition and dTALE2-EBE.

**Supporting Figure 2**. **β-glucuronidase (GUS) staining in *ZjPCK_pro_*:dsRed-STAP1:GUS rice transformants.** **A)** Schematic of the construct used for *Agrobacterium*-mediated transformation in rice. **B)** GUS activity in ten *ZjPCK_pro_*:dsRed-STAP1:GUS transgenic lines. Cross sections (left) and paradermal view (right) of leaves from T_0_ single insertion rice lines show no GUS activity with any of the ten STAPs after staining at 37 ^o^C for 21 h. Scale bars = 100 µm.


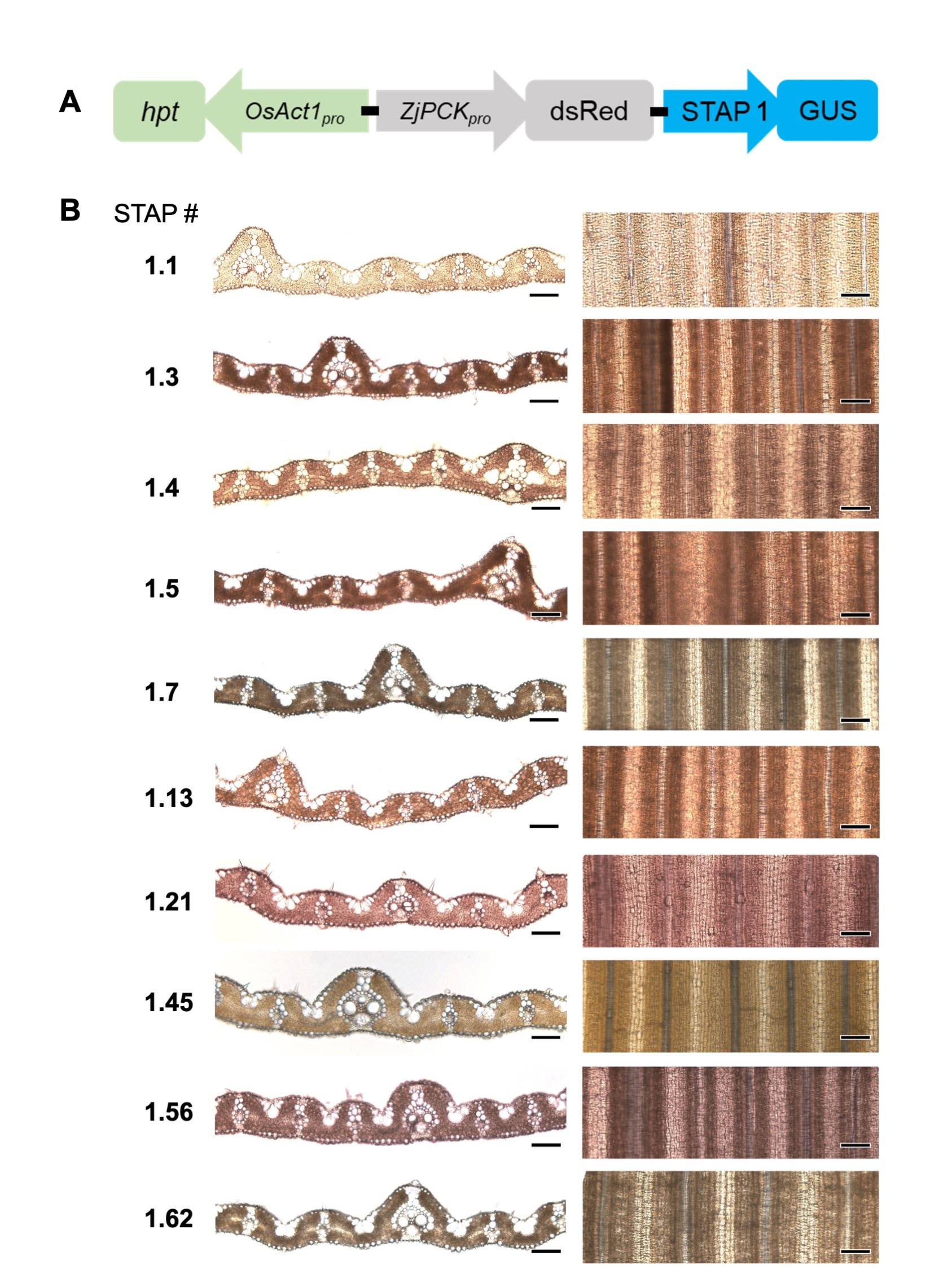


**Supporting Figure 3**. **β-glucuronidase (GUS) activity in *ZmPEPC321_pro_*:mTurquoise-STAP2:GUS rice transformants.** **A)** Schematic of the construct used for *Agrobacterium*-mediated transformation in rice. **B)** GUS activity in seven *ZmPEPC321_pro_*:mTurquoise-STAP2:GUS lines. Cross sections of leaves from T_0_ single insertion rice lines show no GUS activity with any of the seven STAPs after staining at 37^o^ C for 21 h. Scale bars = 50 µm.


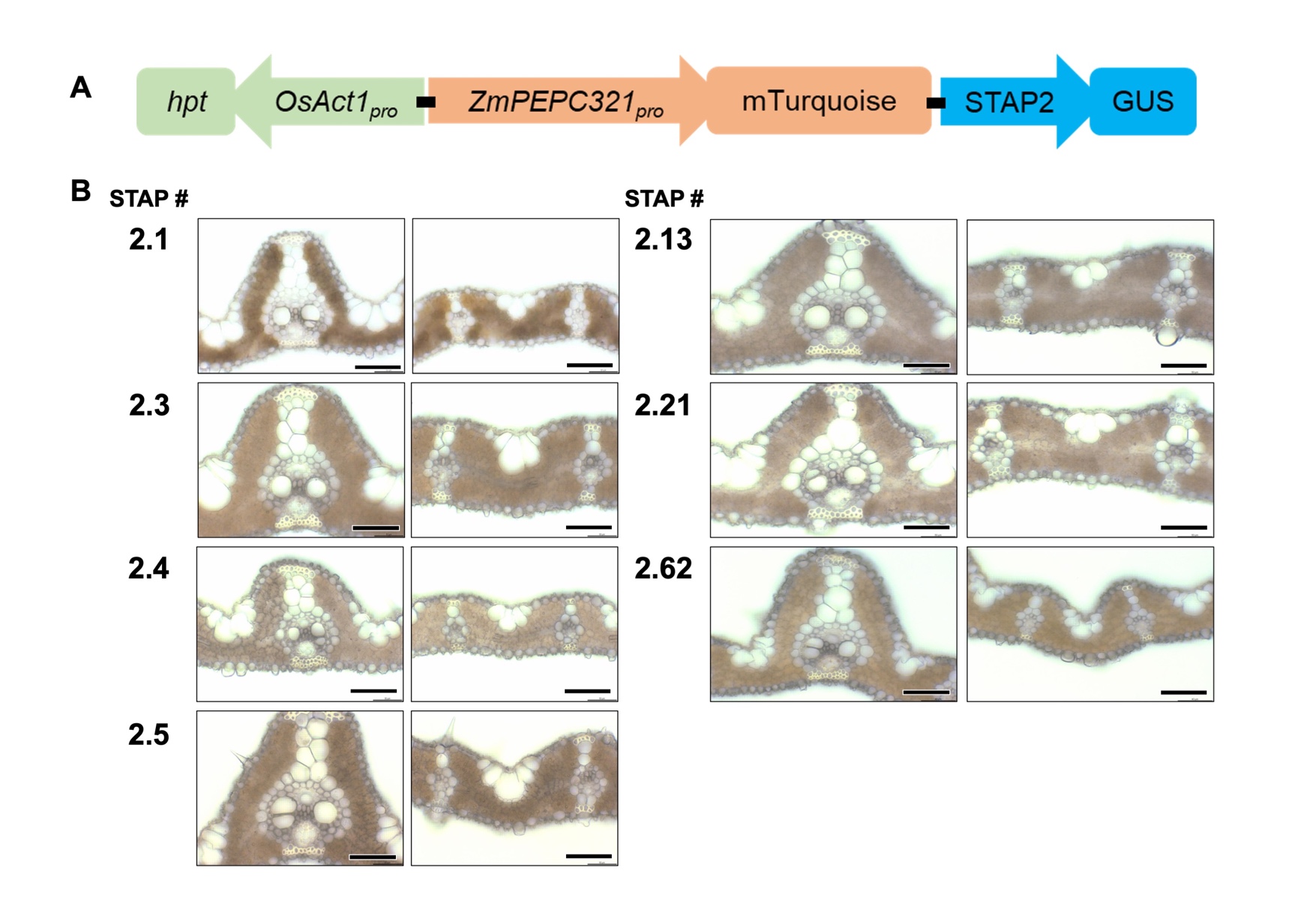


**Supporting Figure 4. Quantification of bundle sheath and mesophyll cell area.** Tiled images of transverse leaf sections from leaf 4 of three wild-type Kitaake plants were used to estimate bundle sheath and mesophyll cell area as a percentage of the total leaf area. Areas were measured using ImageJ.


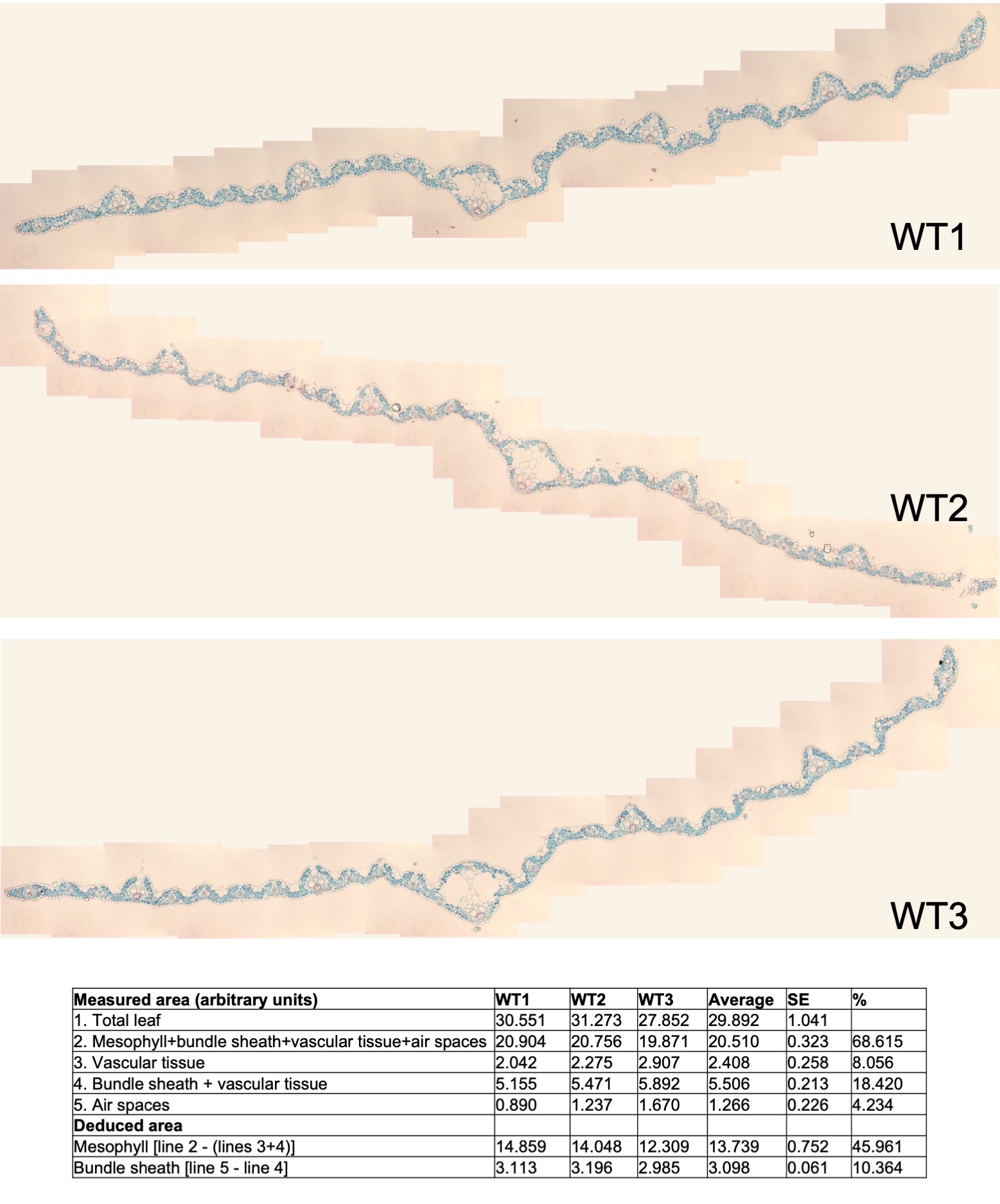


**Supporting Figure 5.** **Linear regression and ANOVA analyses to determine what proportion of variance in GUS transcript abundance in *ZjPCK_pro_*:dTALE1-STAP1-GUS lines was attributable to different factors. A)** The linear regression variables and results for the interaction between dTALE1 mRNA abundance in T_2_ plants and the construct containing the gene, the T_0_ founder plant, and the T_1_ founder plant from which the T_2_ plants were descended. **B)** as in A but for GUS mRNA abundance in T_2_ plants with the inclusion of dTALE1 mRNA abundance as an explanatory (independent) variable. **C)** as in B but for *hpt* mRNA abundance in T_2_ plants. **D)** As in A but for T_1_ founder plant corrected dTALE1 mRNA abundance. **E)** As in B but for T_1_ founder plant corrected GUS mRNA abundance. **F)** As in B but for T_1_ founder plant corrected *hpt* mRNA abundance. **G)** The ANOVA Type II test results for the dependent and explanatory variables listed.

**Supporting Figure 6. Maximum likelihood phylogenetic tree of the orthogroup containing OsKitaake06g213800.** The tree is a consensus tree of a 1000 bootstrap replicate phylogenetic inference computed from the full multiple sequence alignment (no trimming) using IQTREE. The best-fit model of sequence evolution (JTT+I+G4) was inferred automatically. Scale bar indicates number of sequence substitutions per site. The rice gene is highlighted in blue font and the *Arabidopsis thaliana* orthologs are highlighted in red font.


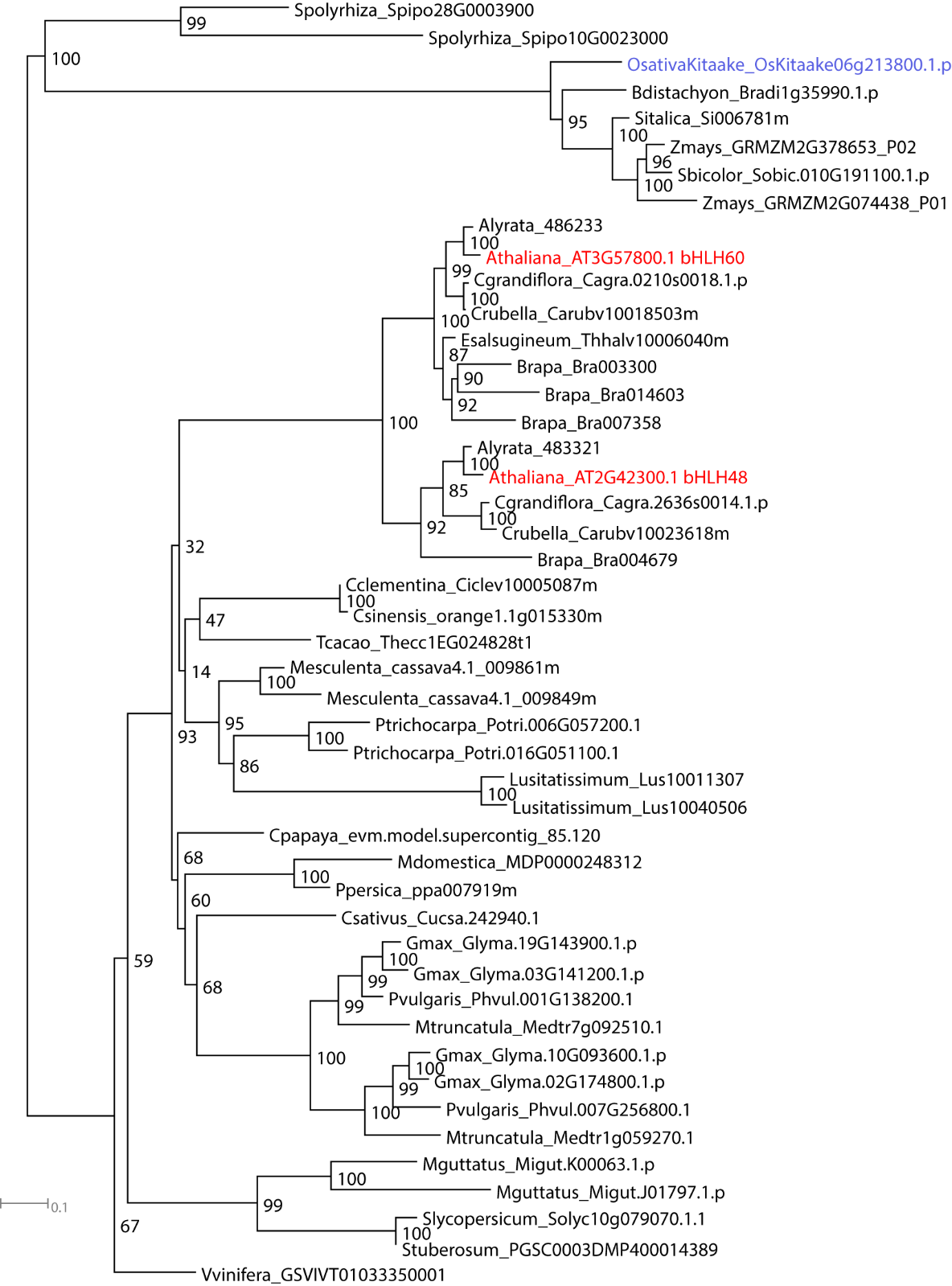


**Supporting Figure 7. Maximum likelihood phylogenetic tree of the orthogroup containing OsKitaake02g392000.** The tree is a consensus tree of a 1000 bootstrap replicate phylogenetic inference computed from the full multiple sequence alignment (no trimming) using IQTREE. The best-fit model of sequence evolution (JTT+I+G4) was inferred automatically. Scale bar indicates number of sequence substitutions per site. The rice gene is highlighted in blue font and the *Arabidopsis thaliana* orthologs are highlighted in red font.


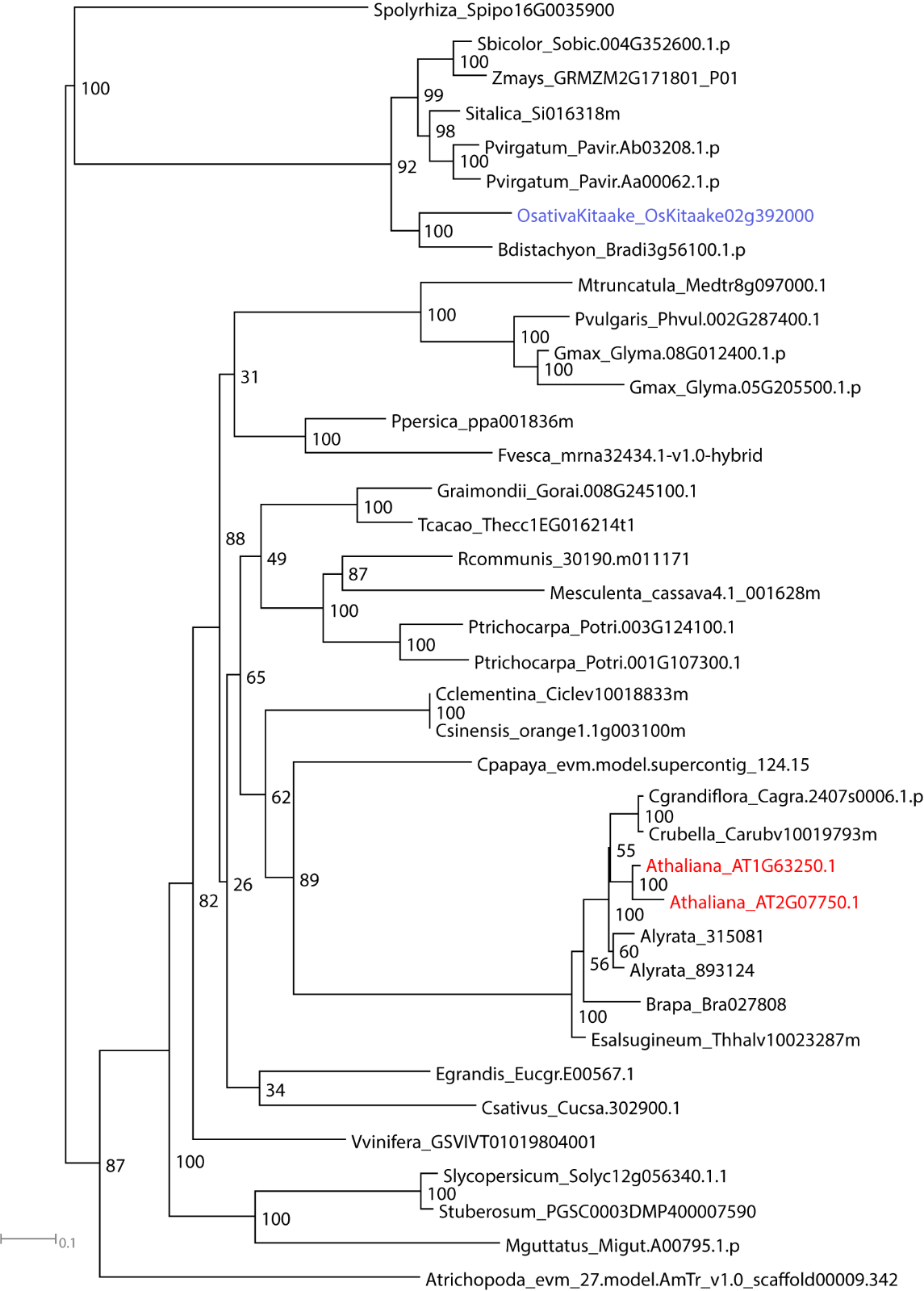


**Supporting Figure 8**. **dTALE and STAP sequences.** **A)** dTALE1-encoding nucleotide sequence. **B)** dTALE1 protein sequence. **C)** dTALE1 repeat variable diresidue (RVD) order and corresponding DNA target sequence (Effector Binding Element - EBE); T_0_ specified by TALE-N-terminal region DNA interaction. **D)** STAP1 nucleotide sequences specific for dTALE1. **E)** Alignment of dTALE1-STAP1 nucleotide sequences. **F)** dTALE2-encoding nucleotide sequence. **G)** dTALE2 protein sequence. **H)** dTALE2 RVD order and corresponding DNA target sequence (Effector Binding Element - EBE); T_0_ specified by TALE-N-terminal region DNA interaction. **I)** STAP2 nucleotide sequences specific for dTALE2. **J)** Alignment of dTALE2-STAP2 nucleotide sequences.

**A.** dTALE1-encoding nucleotide sequence **(**BsaI-flanked Golden Gate Module pAGT2500):

***aatg***GATCCGATTCGTTCTCGGACACCTTCTCCCGCTCGAGAGCTCCTTCCTGGACCACAACCAGATGGAGTGCAACCAACAGCTGATAGGGGTGTATCTCCTCCAGCCGGTGGACCTCTTGACGGCTTACCTGCAAGGCGCACAATGTCCCGTACCAGATTGCCCAGTCCACCAGCACCAAGTCCAGCGTTTTCAGCGGGCAGCTTCTCTGACCTCCTGAGACAGTTTGATCCCTCTTTGTTCAACACCTCACTGTTTGACTCACTTCCGCCTTTTGGGGCTCACCATACTGAAGCCGCTACTGGTGAGTGGGATGAGGTGCAGTCAGGCTTAAGAGCAGCTGATGCCCCACCACCTACGATGAGGGTGGCAGTAACTGCTGCTAGGCCACCGAGAGCTAAACCAGCTCCAAGACGCAGAGCAGCACAACCTTCCGATGCATCTCCTGCTGCTCAAGTCGATCTTCGCACTTTAGGGTATAGCCAGCAACAACAGGAGAAGATCAAACCTAAGGTCCGAAGTACAGTTGCGCAACACCATGAAGCCCTTGTTGGTCATGGGTTCACTCATGCCCACATAGTTGCACTATCCCAACATCCTGCTGCTCTTGGAACTGTTGCGGTGAAGTACCAGGACATGATTGCTGCTTTACCTGAAGCAACACACGAGGCAATAGTCGGTGTTGGCAAACAGTGGTCTGGCGCTAGGGCTCTAGAAGCCCTCCTTACAGTTGCAGGAGAATTGCGGGGACCTCCCTTGCAGCTCGATACCGGACAATTGCTGAAGATTGCCAAACGTGGTGGTGTTACGGCAGTAGAAGCAGTGCATGCTTGGAGGAATGCTCTAACTGGAGCACCCTTGAATCTCACGCCAGAACAAGTGGTCGCTATCGCCTCCCATGATGGTGGAAAACAAGCACTAGAAACTGTCCAAAGATTATTGCCTGTTCTTTGTCAGGCACACGGACTTACCCCACAACAAGTCGTTGCTATAGCCTCCCATGATGGAGGGAAGCAAGCGTTAGAAACAGTGCAGCGGCTACTCCCTGTATTATGCCAGGCTCATGGTCTAACTCCACAACAAGTGGTGGCTATAGCCTCACACGATGGTGGTAAACAGGCACTTGAAACCGTCCAAAGACTCCTGCCGGTCCTCTGCCAGGCACACGGCCTCACCCCCGAACAAGTGGTGGCTATTGCTTCGCATGATGGAGGTAAGCAGGCTTTAGAGACAGTCCAGAGACTACTACCCGTTCTATGCCAGGCCCATGGTTTGACCCCGGAACAGGTTGTTGCTATTGCGTCAAATAATGGCGGCAAGCAAGCGTTGGAAACCGTTCAAGCATTACTCCCTGTTCTCTGTCAAGCACATGGGCTAACGCCCGAGCAGGTTGTTGCAATTGCATCACATGATGGAGGAAAGCAGGCCTTAGAAACGGTACAGGCACTTTTACCAGTCCTTTGCCAAGCACACGGGCTTACACCCGAACAAGTGGTCGCTATTGCAAGTAATATAGGTGGAAAACAAGCACTGGAAACCGTGCAGGCGCTTTTGCCGGTATTATGCCAAGCTCACGGCCTAACTCCTGAACAGGTGGTTGCGATTGCCTCAAATGGTGGGGGTAAACAGGCACTGGAGACTGTGCAGCGGCTTTTGCCTGTTTTGTGTCAAGCTCATGGATTGACACCAGAGCAGGTGGTCGCTATAGCTAGTAACATTGGAGGTAAACAAGCGCTTGAAACCGTGCAACGTCTGCTGCCAGTTCTATGTCAAGCTCATGGGTTGACCCCACAACAGGTTGTAGCGATCGCTTCCAATAACGGAGGAAAGCAAGCTCTAGAAACGGTGCAGAGGCTCCTCCCGGTTCTTTGTCAGGCGCATGGATTGACCCCGGAGCAGGTGGTCGCAATCGCCAGTCATGATGGAGGTAAGCAGGCCTTGGAAACCGTTCAGGCGTTACTCCCGGTTCTATGCCAGGCGCATGGCCTGACCCCTGAACAGGTTGTGGCGATAGCCAGTAACGGCGGGGGAAAGCAGGCACTTGAAACCGTACAACGACTCCTCCCAGTCCTTTGTCAAGCCCACGGATTGACTCCAGAACAAGTAGTTGCTATAGCTTCGAATAATGGAGGAAAGCAGGCCCTTGAAACAGTTCAGCGTCTTTTGCCAGTGTTGTGTCAAGCACACGGATTGACTCCTGAACAGGTTGTCGCCATTGCATCTAATATCGGTGGTAAGCAAGCTCTCGAAACCGTACAGCGACTCTTGCCTGTTCTATGCCAAGCGCATGGCTTGACGCCGGAACAGGTGGTAGCCATAGCAAGCAACATAGGTGGCAAACAAGCTCTTGAAACAGTTCAAAGGTTGTTACCTGTGCTTTGCCAAGCCCACGGTTTGACCCCTCAACAGGTGGTTGCTATAGCATCACATGATGGGGGACGGCCTGCTCTTGAGACAGTGCAGCGCCTGTTGCCCGTGTTGTGTCAAGCGCATGGCTTAACACCGGAACAGGTCGTGGCAATTGCGTCAAATATTGGCGGCAAACAAGCGCTGGAAACCGTTCAGCGACTCTTGCCTGTTCTGTGCCAAGCTCACGGTCTGACGCCCCAACAGGTTGTTGCCATTGCTTCAAATGGAGGAGGGAGGCCAGCCCTTGAGTCGATTGTCGCACAGCTATCTCGGCCCGACCCTGCTTTAGCCGCTCTGACAAATGATCATCTTGTGGCTCTCGCCTGCTTAGGAGGTCGCCCAGCTTTAGACGCAGTAAAAAAGGGTCTACCTCATGCTCCGGCCTTAATCAAGAGGACGAATCGTAGAATCCCAGAACGAACGAGCCATCGCGTAGCCGATCACGCTCAAGTTGTTAGGGTTTTAGGTTTTTTTCAGTGTCATTCACATCCGGCACAAGCTTTCGATGATGCCATGACCCAGTTTGGTATGTCAAGGCATGGATTACTGCAACTTTTCAGAAGAGTAGGAGTGACAGAGCTCGAAGCCAGAAGCGGAACTCTGCCACCCGCTAGCCAAAGATGGGATAGGATATTGCAGGCGAGTGGAATGAAGCGCGCGAAACCATCTCCAACAAGCACTCAAACCCCGGATCAAGCGAGTTTGCACGCTTTCGCAGATTCTCTCGAACGAGATTTGGATGCCCCTTCTCCAATGCACGAAGGTGATCAAACTAGGGCGAGTAGCAGGAAGAGGTCTAGGAGTGATCGTGCAGTTACGGGCCCCTCAGCACAACAGTCTTTTGAGGTCAGGGTGCCAGAACAAAGGGACGCTTTACATCTCCCATTGTCTTGGCGTGTAAAAAGGCCGCGAACTAGTATTGGAGGGGGATTACCGGACCCAGGGACCCCCACTGCTGCTGATCTAGCTGCTTCTAGTACGGTAATGCGCGAGCAAGACGAGGATCCATTTGCTGGGGCAGCTGATGACTTCCCCGCATTCAACGAAGAAGAATTAGCATGGTTGATGGAGTTACTGCCACAGTAA***gctt***

**B.** dTALE1 protein sequence:

MDPIRSRTPSPARELLPGPQPDGVQPTADRGVSPPAGGPLDGLPARRTMSRTRLPSPPAPSPAFSAGSFSDLLRQFDPSLFNTSLFDSLPPFGAHHTEAATGEWDEVQSGLRAADAPPPTMRVAVTAARPPRAKPAPRRRAAQPSDASPAAQVDLRTLGYSQQQQEKIKPKVRSTVAQHHEALVGHGFTHAHIVALSQHPAALGTVAVKYQDMIAALPEATHEAIVGVGKQWSGARALEALLTVAGELRGPPLQLDTGQLLKIAKRGGVTAVEAVHAWRNALTGAPLN

LTPEQVVAIAS**HD**GGKQALETVQRLLPVLCQAHG

LTPQQVVAIAS**HD**GGKQALETVQRLLPVLCQAHG

LTPQQVVAIAS**HD**GGKQALETVQRLLPVLCQAHG

LTPEQVVAIAS**HD**GGKQALETVQRLLPVLCQAHG

LTPEQVVAIAS**NN**GGKQALETVQALLPVLCQAHG

LTPEQVVAIAS**HD**GGKQALETVQALLPVLCQAHG

LTPEQVVAIAS**NI**GGKQALETVQALLPVLCQAHG

LTPEQVVAIAS**NG**GGKQALETVQRLLPVLCQAHG

LTPEQVVAIAS**NI**GGKQALETVQRLLPVLCQAHG

LTPQQVVAIAS**NN**GGKQALETVQRLLPVLCQAHG

LTPEQVVAIAS**HD**GGKQALETVQALLPVLCQAHG

LTPEQVVAIAS**NG**GGKQALETVQRLLPVLCQAHG

LTPEQVVAIAS**NN**GGKQALETVQRLLPVLCQAHG

LTPEQVVAIAS**NI**GGKQALETVQRLLPVLCQAHG

LTPEQVVAIAS**NI**GGKQALETVQRLLPVLCQAHG

LTPQQVVAIAS**HD**GGRPALETVQRLLPVLCQAHG

LTPEQVVAIAS**NI**GGKQALETVQRLLPVLCQAHG

LTPQQVVAIAS**NG**GGRPALE

SIVAQLSRPDPALAALTNDHLVALACLGGRPALDAVKKGLPHAPALIKRTNRRIPERTSHRVADHAQVVRVLGFFQCHSHPAQAFDDAMTQFGMSRHGLLQLFRRVGVTELEARSGTLPPASQRWDRILQASGMKRAKPSPTSTQTPDQASLHAFADSLERDLDAPSPMHEGDQTRASSRKRSRSDRAVTGPSAQQSFEVRVPEQRDALHLPLSWRVKRPRTSIGGGLPDPGTPTAADLAASSTVMREQDEDPFAGAADDFPAFNEEELAWLMELLPQ*

**C.** dTALE1 RVD-order and corresponding EBE; T_0_ specified by TALE-N-terminal region DNA interaction:

**RVDS: HD HD HD HD NN HD NI NG NI NN HD NG NN NI NI HD NI NG**

EBE T0 C C C C G C A T A G C T G A A C A T

**D.** Corresponding orthogonal STAP1s for dTALE1:

STAP1.1

***ggag***GTTATCACACTTGGTAATT**TCCCCGCATAGCTGAACAT**CTATACAACACTCATCGCGCGGAGAAACGCGCAACAAATTGGAGGCGCATT***aatg***

STAP1.3

***ggag***GTCGTCAAATGATAAGTAC**TCCCCGCATAGCTGAACAT**CTATATAACGCAGCAAATGGTTGAAACGTATTAGCAACAGCTAAACCTTCG***aatg***

STAP1.4

***ggag***GCGTGTCGTTTTAGTGAGG**TCCCCGCATAGCTGAACAT**CTATATAAGGTTTTGCTATTCATTGAAAGCAGTAGTGACTGATTTGTATAT***aatg***

STAP1.5

***ggag***GTTCTGTGGGTTTTTTGGG**TCCCCGCATAGCTGAACAT**CTATATAATGAGAAACAGGGGCAAATCCCCGAAAACAAATAACGCGAAGC***aatg***

STAP1.13

***ggag***GTAAGTTCTGGCCTCTGGG**TCCCCGCATAGCTGAACAT**CTATATAATAGGACAGTAAGATATGCCAGACAATGTAGCACGTAGAACGGA***aatg***

STAP1.21

***ggag***GCCGTCGGGGGGGGGTCTG**TCCCCGCATAGCTGAACAT**CTATATAATACCGGTTATCCAGCGACAACCGTCAATAACTTAACATTGAAC***aatg***

STAP1.45

***ggag***TCAGAGTGTGGTCGTGTTT**TCCCCGCATAGCTGAACAT**CTATATAAGTTTACACCTGCCCGACCTATTCCTTGATGAGCTCGCGCACCG***aatg***

STAP1.56

***ggag***ATATTCCTCGGCTGTCACT**TCCCCGCATAGCTGAACAT**CTATATAATCAAATTCCTAGGGGCTGCCATGGTTACCCCTTGCTATGTAGC***aatg***

STAP1.62

***ggag***TATTGGTCCACTTGCTCCG**TCCCCGCATAGCTGAACAT**CTATATAACGCAGAAACCTCGCAACTGACGTTCCGATTGCGATAAACACAC***aatg***

**E.** Alignment of dTALE1-STAP1 nucleotide sequences:


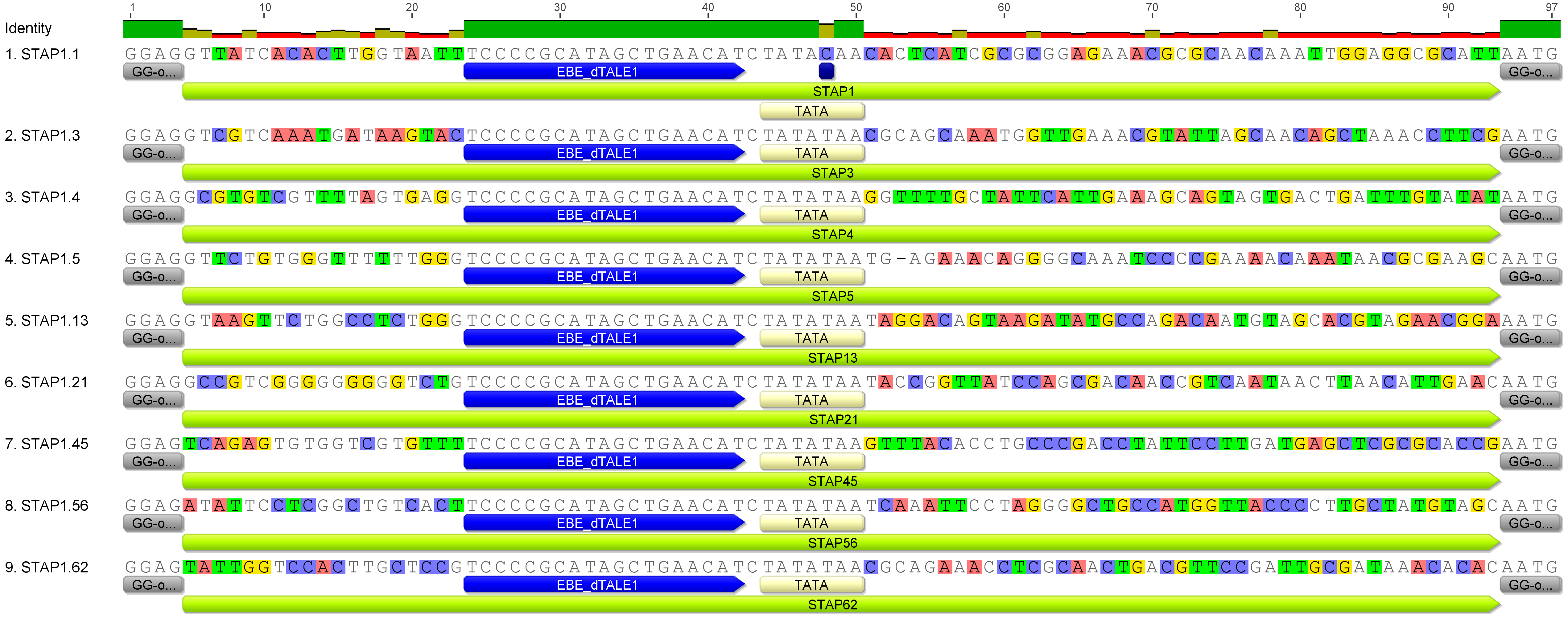


**F.** dTALE2-encoding nucleotide sequence (BsaI-flanked Golden Gate Module pAGT5805):

***aatg***GATCCGATTCGTTCTCGGACACCTTCTCCCGCTCGAGAGCTCCTTCCTGGACCACAACCAGATGGAGTGCAACCAACAGCTGATAGGGGTGTATCTCCTCCAGCCGGTGGACCTCTTGACGGCTTACCTGCAAGGCGCACAATGTCCCGTACCAGATTGCCCAGTCCACCAGCACCAAGTCCAGCGTTTTCAGCGGGCAGCTTCTCTGACCTCCTGAGACAGTTTGATCCCTCTTTGTTCAACACCTCACTGTTTGACTCACTTCCGCCTTTTGGGGCTCACCATACTGAAGCCGCTACTGGTGAGTGGGATGAGGTGCAGTCAGGCTTAAGAGCAGCTGATGCCCCACCACCTACGATGAGGGTGGCAGTAACTGCTGCTAGGCCACCGAGAGCTAAACCAGCTCCAAGACGCAGAGCAGCACAACCTTCCGATGCATCTCCTGCTGCTCAAGTCGATCTTCGCACTTTAGGGTATAGCCAGCAACAACAGGAGAAGATCAAACCTAAGGTCCGAAGTACAGTTGCGCAACACCATGAAGCCCTTGTTGGTCATGGGTTCACTCATGCCCACATAGTTGCACTATCCCAACATCCTGCTGCTCTTGGAACTGTTGCGGTGAAGTACCAGGACATGATTGCTGCTTTACCTGAAGCAACACACGAGGCAATAGTCGGTGTTGGCAAACAGTGGTCTGGCGCTAGGGCTCTAGAAGCCCTCCTTACAGTTGCAGGAGAATTGCGGGGACCTCCCTTGCAGCTCGATACCGGACAATTGCTGAAGATTGCCAAACGTGGTGGTGTTACGGCAGTAGAAGCAGTGCATGCTTGGAGGAATGCTCTAACTGGAGCACCCTTGAATCTCACGCCAGAACAAGTGGTCGCTATCGCCTCCAATCATGGTGGAAAACAAGCACTAGAAACTGTCCAAAGATTATTGCCTGTTCTTTGTCAGGCACACGGACTTACCCCACAACAAGTCGTTGCTATAGCCTCCAATATCGGAGGGAAGCAAGCGTTAGAAACAGTGCAGCGGCTACTCCCTGTATTATGCCAGGCTCATGGTCTAACTCCACAACAAGTGGTGGCTATAGCCTCACACGATGGTGGTAAACAGGCACTTGAAACCGTCCAAAGACTCCTGCCGGTCCTCTGCCAGGCACACGGCCTCACCCCCGAACAAGTGGTGGCTATTGCTTCGAATCACGGAGGTAAGCAGGCTTTAGAGACAGTCCAGAGACTACTACCCGTTCTATGCCAGGCCCATGGTTTGACCCCGGAACAGGTTGTTGCTATTGCGTCACACGATGGCGGCAAGCAAGCGTTGGAAACCGTTCAAGCATTACTCCCTGTTCTCTGTCAAGCACATGGGCTAACGCCCGAGCAGGTTGTTGCAATTGCATCAAATAACGGAGGAAAGCAGGCCTTAGAAACGGTACAGGCACTTTTACCAGTCCTTTGCCAAGCACACGGGCTTACACCCGAACAAGTGGTCGCTATTGCAAGTCATGATGGTGGAAAACAAGCACTGGAAACCGTGCAGGCGCTTTTGCCGGTATTATGCCAAGCTCACGGCCTAACTCCTGAACAGGTGGTTGCGATTGCCTCAAATCATGGGGGTAAACAGGCACTGGAGACTGTGCAGCGGCTTTTGCCTGTTTTGTGTCAAGCTCATGGATTGACACCAGAGCAGGTGGTCGCTATAGCTAGTAACATTGGAGGTAAACAAGCGCTTGAAACCGTGCAACGTCTGCTGCCAGTTCTATGTCAAGCTCATGGGTTGACCCCACAACAGGTTGTAGCGATCGCTTCCAATGGCGGAGGAAAGCAAGCTCTAGAAACGGTGCAGAGGCTCCTCCCGGTTCTTTGTCAGGCGCATGGATTGACCCCGGAGCAGGTGGTCGCAATCGCCAGTAATATAGGAGGTAAGCAGGCCTTGGAAACCGTTCAGGCGTTACTCCCGGTTCTATGCCAGGCGCATGGCCTGACCCCTGAACAGGTTGTGGCGATAGCCAGTAACCATGGGGGAAAGCAGGCACTTGAAACCGTACAACGACTCCTCCCAGTCCTTTGTCAAGCCCACGGATTGACTCCAGAACAAGTAGTTGCTATAGCTTCGAATGGAGGAGGAAAGCAGGCCCTTGAAACAGTTCAGCGTCTTTTGCCAGTGTTGTGTCAAGCACACGGATTGACTCCTGAACAGGTTGTCGCCATTGCATCTAATGGTGGTGGTAAGCAAGCTCTCGAAACCGTACAGCGACTCTTGCCTGTTCTATGCCAAGCGCATGGCTTGACGCCGGAACAGGTGGTAGCCATAGCAAGCAACGGTGGTGGCAAACAAGCTCTTGAAACAGTTCAAAGGTTGTTACCTGTGCTTTGCCAAGCCCACGGTTTGACCCCTCAACAGGTGGTTGCTATAGCATCACATGATGGGGGACGGCCTGCTCTTGAGACAGTGCAGCGCCTGTTGCCCGTGTTGTGTCAAGCGCATGGCTTAACACCGGAACAGGTCGTGGCAATTGCGTCACACGATGGCGGCAAACAAGCGCTGGAAACCGTTCAGCGACTCTTGCCTGTTCTGTGCCAAGCTCACGGTCTGACGCCCCAACAGGTTGTTGCCATTGCTTCAAATATTGGAGGGAGGCCAGCCCTTGAGTCGATTGTCGCACAGCTATCTCGGCCCGACCCTGCTTTAGCCGCTCTGACAAATGATCATCTTGTGGCTCTCGCCTGCTTAGGAGGTCGCCCAGCTTTAGACGCAGTAAAAAAGGGTCTACCTCATGCTCCGGCCTTAATCAAGAGGACGAATCGTAGAATCCCAGAACGAACGAGCCATCGCGTAGCCGATCACGCTCAAGTTGTTAGGGTTTTAGGTTTTTTTCAGTGTCATTCACATCCGGCACAAGCTTTCGATGATGCCATGACCCAGTTTGGTATGTCAAGGCATGGATTACTGCAACTTTTCAGAAGAGTAGGAGTGACAGAGCTCGAAGCCAGAAGCGGAACTCTGCCACCCGCTAGCCAAAGATGGGATAGGATATTGCAGGCGAGTGGAATGAAGCGCGCGAAACCATCTCCAACAAGCACTCAAACCCCGGATCAAGCGAGTTTGCACGCTTTCGCAGATTCTCTCGAACGAGATTTGGATGCCCCTTCTCCAATGCACGAAGGTGATCAAACTAGGGCGAGTAGCAGGAAGAGGTCTAGGAGTGATCGTGCAGTTACGGGCCCCTCAGCACAACAGTCTTTTGAGGTCAGGGTGCCAGAACAAAGGGACGCTTTACATCTCCCATTGTCTTGGCGTGTAAAAAGGCCGCGAACTAGTATTGGAGGGGGATTACCGGACCCAGGGACCCCCACTGCTGCTGATCTAGCTGCTTCTAGTACGGTAATGCGCGAGCAAGACGAGGATCCATTTGCTGGGGCAGCTGATGACTTCCCCGCATTCAACGAAGAAGAATTAGCATGGTTGATGGAGTTACTGCCACAGTAA***gctt***

**G.** dTALE2 protein sequence:

MDPIRSRTPSPARELLPGPQPDGVQPTADRGVSPPAGGPLDGLPARRTMSRTRLPSPPAPSPAFSAGSFSDLLRQFDPSLFNTSLFDSLPPFGAHHTEAATGEWDEVQSGLRAADAPPPTMRVAVTAARPPRAKPAPRRRAAQPSDASPAAQVDLRTLGYSQQQQEKIKPKVRSTVAQHHEALVGHGFTHAHIVALSQHPAALGTVAVKYQDMIAALPEATHEAIVGVGKQWSGARALEALLTVAGELRGPPLQLDTGQLLKIAKRGGVTAVEAVHAWRNALTGAPLN

LTPEQVVAIAS**NH**GGKQALETVQRLLPVLCQAHG

LTPQQVVAIAS**NI**GGKQALETVQRLLPVLCQAHG

LTPQQVVAIAS**HD**GGKQALETVQRLLPVLCQAHG

LTPEQVVAIAS**NH**GGKQALETVQRLLPVLCQAHG

LTPEQVVAIAS**HD**GGKQALETVQALLPVLCQAHG

LTPEQVVAIAS**NN**GGKQALETVQALLPVLCQAHG

LTPEQVVAIAS**HD**GGKQALETVQALLPVLCQAHG

LTPEQVVAIAS**NH**GGKQALETVQRLLPVLCQAHG

LTPEQVVAIAS**NI**GGKQALETVQRLLPVLCQAHG

LTPQQVVAIAS**NG**GGKQALETVQRLLPVLCQAHG

LTPEQVVAIAS**NI**GGKQALETVQALLPVLCQAHG

LTPEQVVAIAS**NH**GGKQALETVQRLLPVLCQAHG

LTPEQVVAIAS**NG**GGKQALETVQRLLPVLCQAHG

LTPEQVVAIAS**NG**GGKQALETVQRLLPVLCQAHG

LTPEQVVAIAS**NG**GGKQALETVQRLLPVLCQAHG

LTPQQVVAIAS**HD**GGRPALETVQRLLPVLCQAHG

LTPEQVVAIAS**HD**GGKQALETVQRLLPVLCQAHG

LTPQQVVAIAS**NI**GGRPALE

SIVAQLSRPDPALAALTNDHLVALACLGGRPALDAVKKGLPHAPALIKRTNRRIPERTSHRVADHAQVVRVLGFFQCHSHPAQAFDDAMTQFGMSRHGLLQLFRRVGVTELEARSGTLPPASQRWDRILQASGMKRAKPSPTSTQTPDQASLHAFADSLERDLDAPSPMHEGDQTRASSRKRSRSDRAVTGPSAQQSFEVRVPEQRDALHLPLSWRVKRPRTSIGGGLPDPGTPTAADLAASSTVMREQDEDPFAGAADDFPAFNEEELAWLMELLPQ*

**H.** dTALE2 RVD-order and corresponding EBE; T0 specified by TALE-N-terminal region DNA interaction:

**RVDS: NH NI HD NH HD NN HD NH NI NG NI NH NG NG NG HD HD NI**

EBE T0 G A C G C G C G A T A G T T T C C A

**I.** Corresponding orthogonal STAP2s for dTALE2:

STAP2.1

***ggag***GTTATCACACTTGGTAATT**TGACGCGCGATAGTTTCCA**CTATACAACACTCATCGCGCGGAGAAACGCGCAACAAATTGGAGGCGCATT***aatg***

STAP2.3

***ggag***GTCGTCAAATGATAAGTAC**TGACGCGCGATAGTTTCCA**CTATATAACGCAGCAAATGGTTGAAACGTATTAGCAACAGCTAAACCTTCG***aatg***

STAP2.4

***ggag***GCGTGTCGTTTTAGTGAGG**TGACGCGCGATAGTTTCCA**CTATATAAGGTTTTGCTATTCATTGAAAGCAGTAGTGACTGATTTGTATAT***aatg***

STAP2.5

***ggag***GTTCTGTGGGTTTTTTGGG**TGACGCGCGATAGTTTCCA**CTATATAATGAGAAACAGGGGCAAATCCCCGAAAACAAATAACGCGAAGC***aatg***

STAP2.13

***ggag***GTAAGTTCTGGCCTCTGGG**TGACGCGCGATAGTTTCCA**CTATATAATAGGACAGTAAGATATGCCAGACAATGTAGCACGTAGAACGGA***aatg***

STAP2.21

***ggag***GCCGTCGGGGGGGGGTCTG**TGACGCGCGATAGTTTCCA**CTATATAATACCGGTTATCCAGCGACAACCGTCAATAACTTAACATTGAAC***aatg***

STAP2.62

***ggag***TATTGGTCCACTTGCTCCG**TGACGCGCGATAGTTTCCA**CTATATAACGCAGAAACCTCGCAACTGACGTTCCGATTGCGATAAACACAC***aatg***

**J.** Alignment of dTALE2-STAP2 nucleotide sequences:


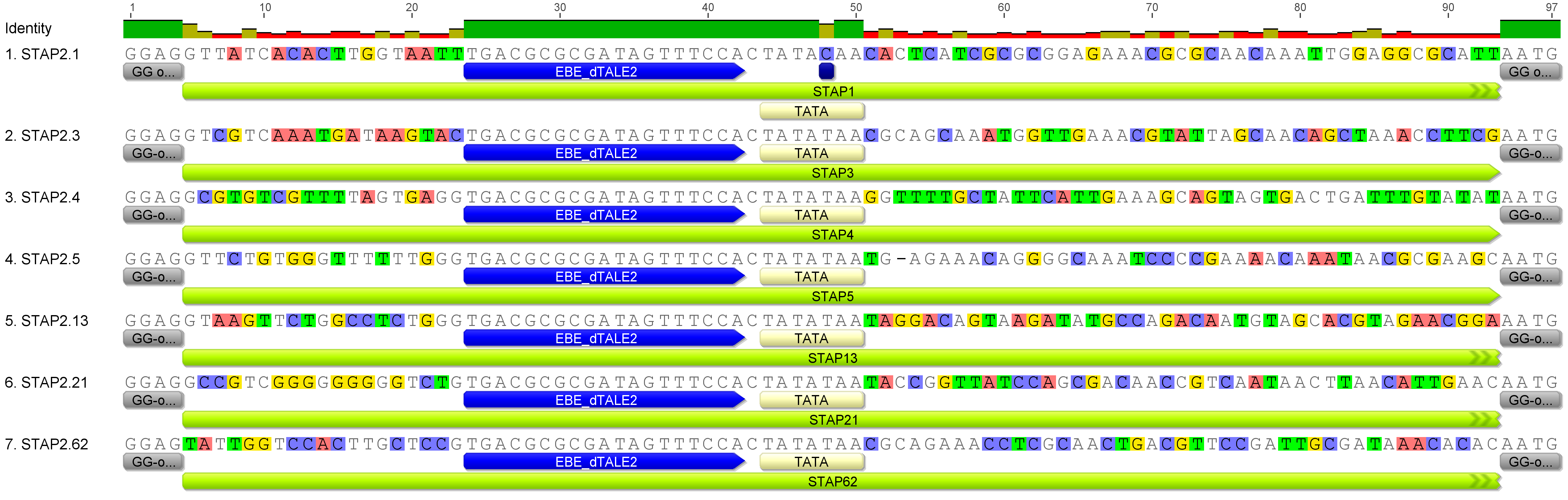

Supplement: Supplementary file 4 — Figure S1 dTALE scoring system and RVD‐composition of rice orthogonal dTALE2. Figure S2 β‐glucuronidase (GUS) staining in ZjPCK pro :dsRed‐STAP1:GUS rice transformants. Figure S3 β‐glucuronidase (GUS) activity in ZmPEPC321 pro :mTurquoise‐STAP2:GUS rice transformants. Figure S4 Quantification of bundle sheath and mesophyll cell area. Figure S5 Linear regression and ANOVA analyses to determine what proportion of variance in GUS transcript abundance in ZjPCK pro :dTALE1‐STAP1‐GUS lines was attributable to different factors. Figure S6 Maximum likelihood phylogenetic tree of the orthogroup containing OsKitaake06g213800. Figure S7 Maximum likelihood phylogenetic tree of the orthogroup containing OsKitaake02g392000. Figure S8 dTALE and STAP sequences. [file PBI-20-1786-s004.docx]
